# Measuring the Level of Aflatoxin Infection in Pistachio Nuts by Applying Machine Learning Techniques to Hyperspectral Images

**DOI:** 10.3390/s25051548

**Published:** 2025-03-02

**Authors:** Lizzie Williams, Pancham Shukla, Akbar Sheikh-Akbari, Sina Mahroughi, Iosif Mporas

**Affiliations:** 1 Department of Computing, Imperial College London, London SW7 2AZ, UK; lizzies.williams02@gmail.com (L.W.); panchamkumar.shukla@imperial.ac.uk (P.S.); 2 School of Built Environment, Engineering and Computing, Leeds Beckett University, Leeds LS6 3QS, UK; s.mahroughi@leedsbeckett.ac.uk; 3 Department of Engineering and Technology, School of Physics, Engineering & Computer Science, University of Hertfordshire, Hatfield AL10 9AB, UK; i.mporas@herts.ac.uk

**Keywords:** pistachios, aflatoxin, hyperspectral imaging, residual networks, k-means clustering, dimensionality reduction, machine learning

## Abstract

This paper investigates the use of machine learning techniques on hyperspectral images of pistachios to detect and classify different levels of aflatoxin contamination. Aflatoxins are toxic compounds produced by moulds, posing health risks to consumers. Current detection methods are invasive and contribute to food waste. This paper explores the feasibility of a non-invasive method using hyperspectral imaging and machine learning to classify aflatoxin levels accurately, potentially reducing waste and enhancing food safety. Hyperspectral imaging with machine learning has shown promise in food quality control. The paper evaluates models including Dimensionality Reduction with K-Means Clustering, Residual Networks (ResNets), Variational Autoencoders (VAEs), and Deep Convolutional Generative Adversarial Networks (DCGANs). Using a dataset from Leeds Beckett University with 300 hyperspectral images, covering three aflatoxin levels (<8 ppn, >160 ppn, and >300 ppn), key wavelengths were identified to indicate contamination presence. Dimensionality Reduction with K-Means achieved 84.38% accuracy, while a ResNet model using the 866.21 nm wavelength reached 96.67%. VAE and DCGAN models, though promising, were constrained by dataset size. The findings highlight the potential for machine learning-based hyperspectral imaging in pistachio quality control, and future research should focus on expanding datasets and refining models for industry application.

## 1. Introduction

Pistachios are a type of tree nut, which are susceptible to contamination from aflatoxins. Aflatoxins are produced by fungal moulds, most commonly Aspergillus flavus and Aspergillus parasiticus [1]. The consumption of pistachios with high levels of aflatoxins can be harmful to consumers, increasing their risks of developing liver cancer significantly [2]. Therefore, pistachio suppliers must ensure that their pistachios conform to aflatoxin regulations. The UK currently states that pistachios intended for direct human consumption or to be used as an ingredient in food products must have a maximum level of 8 μg/kg of aflatoxin B1 and a maximum level of 10 μg/kg of the sum of aflatoxins B1, B2, G1, and G2 [3].

Farmers currently use invasive methods to test the aflatoxin levels within pistachios, involving finely grounding a sample of pistachios to use for assessment [1]. Therefore, the pistachios from a sample used for testing are no longer able to be sold or consumed, leading to a lot of waste. The entire sample which is identified to have levels of aflatoxin above the regulations is also disposed of. The introduction of a multispectral camera on the production line would allow for pistachios tested to still be sold and consumed if they conform to regulations. It would also provide the opportunity to test more frequently in smaller batches to reduce the amount of pistachios disposed of when a sample is identified as being above the limit.

Hyperspectral imaging is a technique used to gain spatial and spectral information on the intended target simultaneously. This information can be used for detection within each pixel of a hyperspectral image [4]. By capturing spectral signals linked to degradation, HSI makes high-volume yet non-destructive scanning possible and facilitates identification ahead of consumer exposure to it. HSI is capable of accurately distinguishing between acceptable and unsafe food products [5] by analysing the different spectral absorption and visibility properties of contaminated samples. Advanced machine learning algorithms can automate the extraction and classification of features, lowering the requirement for manual inspection. This improves the method’s effectiveness when combined with neural network models [6]. By providing real-time food safety monitoring, HSI may mitigate monetary losses while significantly improving contamination identifying rates. Even with constraints such as substantial data dimensionality and computing needs, HSI has grown into an increasingly beneficial instrument for large-scale food safety tasks like aflatoxin level quantification, aligning with ongoing advancements in hardware acceleration, feature selection, and machine learning. This study aims towards improving contemporary methods by developing ML models for more accurate and efficient aflatoxin contamination level classification using HSI data.

**Figure 1:**
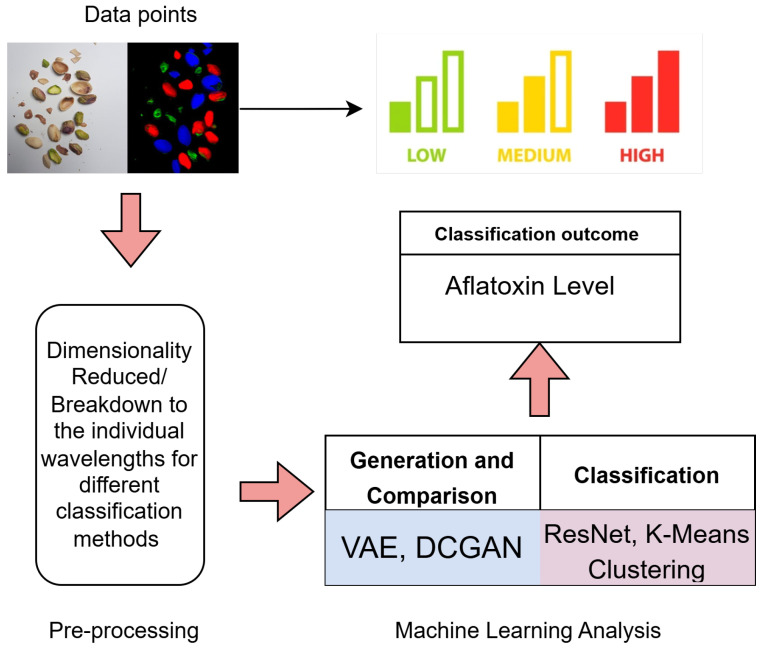
Classification system implemented in this research for each of the classifiers developed. For ResNet, VAE, and DCGAN, the hyperspectral images are broken down into individual wavelengths but for K-Means Clustering, the images have their dimensionality reduced.

Hence, this paper uses hyperspectral images of pistachios to create models which can classify the samples’ aflatoxin levels from only a single wavelength of each image. It evaluates Residual Networks (ResNets), Variational Autoencoders (VAEs), and Deep Convolutional Generative Adversarial Networks (DCGANs) and compares their performance to Dimensionality Reduction with K-Means Clustering used on an entire hyperspectral image. The overall system structure can been seen in Figure 1.

If the models based on a few wavelengths have the ability to correctly classify the level of aflatoxin within the pistachios, then a multispectral camera could be used with a machine learning model to classify the aflatoxin level of a sample of pistachios. This could be used to simplify the aflatoxin testing process for pistachio farmers to increase their profits, decrease the amount of wasted pistachios, and hopefully reduce the number of toxic pistachios consumed.

There are also some drawbacks to take into consideration. The need for a good amount of data is a key drawback, as a prerequisite for machine learning models to train effectively. They typically need large, labelled datasets, and small sample numbers can result in overfitting. The handling of hyperspectral images is computationally challenging and requires significant resources and time, particularly when using advanced algorithms such as deep learning and computer vision. Moreover, deep learning models behave as “black boxes”, which makes it difficult to understand how they make conclusions. Hyperspectral data are also sensitive to noise and the surrounding environment, which can affect detection accuracy if not administered appropriately.

This paper addresses the limitations of these approaches for hyperspectral imaging by employing a dataset of 300 images, performing data augmentation to increase model generalisation and minimise overfitting. We adopted dimensionality reduction techniques to reduce data dimensions and increase computing speed. Our findings additionally show that it is reasonable to integrate these models into conventional food safety procedures for immediate quality control.

After further work, the final implemented model would be used to ensure pistachios tested were of a suitable level in order to reduce the chances of consumers developing illnesses. Consequently, helping to develop a more effective method could help to decrease the number of people poisoned by aflatoxins, benefiting the health of pistachio consumers. If the model were to be faulty, then the research could have the opposite affect.

In summary, this paper explores the use of hyperspectral imaging to simplify the quantification process of aflatoxin contamination levels for pistachio suppliers. The paper makes several contributions to the pistachio nut industry by using techniques that have never been applied to hyperspectral images of pistachios, which are not labelled pixel-wise. In particular, the paper:

1. Analyses and identifies the important wavelengths for effective detection of aflatoxins within pistachio nuts. The key wavelengths were identified as 399.98 nm, 584.64 nm, 704.64 nm, 866.21 nm, and 1002.23 nm.
2. Rigorously evaluates multiple classifiers using only important wavelengths, such as 866.21 nm, to determine aflatoxin levels, demonstrating the effectiveness of ResNet with a 96.67% accuracy at wavelength 866.21 nm.
3. Applies the unsupervised learning approach Dimensionality Reduction combined with K-Means Clustering to the entire hyperspectral image dataset to classify the images, thereby improving the computational time complexity of the K-Means algorithm through dimensionality reduction by at least 34,151.04 times.

## 2. Background

This section explores the consumption of pistachio nuts (Section 2.1) and analyses aflatoxin detection within the pistachio industry and the various methods used (Section 2.2). Next, the chapter discusses what hyperspectral imaging is and its applications (Section 2.3).

### 2.1. Pistachio Consumption and Industry

The annual per capita utilisation of pistachio nuts in the United States increased from 0.2 to 0.5 pounds between 2000 to 2018. Meanwhile, the value of the production of pistachio nuts in the United States increased from approximately USD 200 million to over USD 2.5 billion, indicating a radical increase in consumption [7].

The rise in pistachio nut consumption is likely to be due to an increased awareness of the nut’s health benefits. Pistachios contain healthy fats, lots of beneficial minerals, vitamins, and phenolic compounds, which have antioxidant and anti-inflammatory properties, helping to reduce the risk of certain types of diseases which involve overproduction [8]. These increasing production numbers not only indicate there are more consumers experiencing these health benefits but also more consumers at risk if the nuts contain aflatoxins. The production companies are also processing larger quantities of pistachio nuts, therefore scaling their costs of aflatoxin detection considerably.

The introduction of a simpler, less invasive aflatoxin detection technique would allow companies to reduce their costs and process pistachios quicker. It may also allow companies to increase the percentage of pistachios being tested, reducing the risk of aflatoxin contamination to consumers.

### 2.2. Aflatoxin Detection Within the Pistachio Industry

There are a variety of methods of aflatoxin detection currently used within the pistachio industry. The method a company uses depends on their financial situation; for example, pistachio suppliers in developing countries more commonly use simpler, less equipment-heavy methods [9].

#### 2.2.1. The Importance of Aflatoxin Detection Within the Pistachio Industry

Aflatoxin B1, the main aflatoxin contaminating pistachios, has been classified as a Group I carcinogen by The International Agency for Research on Cancer (IARC) [10,11]. Therefore, there are maximum tolerated levels (MTLs) for the amount of aflatoxin within pistachio nuts [11]. Within the UK, pistachios intended for direct human consumption or to be used as an ingredient in food products have an MTL of 8 μg/kg for aflatoxin B1 and an MTL of 10 μg/kg for the sum of aflatoxins B1, B2, G1, and G2 [3]. Consequently, the detection of aflatoxins is important to ensure pistachio legislation is followed, which helps to stop consumers becoming ill, and in some cases, developing cancer [11].

#### 2.2.2. Thin-Layer Chromatography

Thin-Layer Chromatography (TLC) was the first method developed and used for detecting aflatoxins [9]. In the 1980s many developed countries switched over to using modern methods, such as HPLC. These methods require more equipment but perform better than TLC [9]. TLC involves using a thin, even sorbent layer, usually between 0.1 and 0.25 mm thick, applied to a support backing, usually made of glass [12]. The sample (in our case, finely ground pistachio [1]) is dissolved in the appropriate solvent and then applied to one side of the sorbent layer approximately 1 cm from the edge [12]. Then, this sorbent layer and support is placed in a tank containing an eluent at a depth which lays just below the sample [12]. The eluent absorbs through the sorbent layer upwards, migrating the sample at different rates to separate it [12]. Once the eluent has migrated to the top of the sorbent layer the layer is dried and evaluated [12]. The aflatoxin levels are most commonly quantified by comparing the samples with standards under a UV light or by densitometric scanning [13].

Despite TLC being a simple, and relatively cost effective method, it has limitations due to the inconsistencies in the mobile-phase of the chromatography [14]. It is hard to control this stage of the method to create consistent results for comparison. Comparison under UV light is also subjective to the person assessing the samples and their experience level, which may lead to inconsistent results [13,14].

#### 2.2.3. High-Performance Liquid Chromatography

High-Performance Liquid Chromatography (HPLC) is a more commonly used method for identifying the levels of aflatoxin in pistachios, specifically within developed countries [9]. A HPLC system usually involves a stationary phase, a mobile phase with varying polarity levels, and an ultra-violet (UV) detector [15].

The system starts in the mobile phase where the solvent continuously flows at a specified, monitored rate using a high-pressure pump. The sample is then injected into the continuous flow of solvent and passes into the HPLC column where the stationary phase takes place. The packing material helps with the separation of the sample. A detector then identifies these separated components as they emerge from the column [15]. These results are then analysed to detect the level of aflatoxin within the samples of pistachios.

HPLC can be combined with a fluorescence detector, instead of a UV detector, for pistachio aflatoxin level detection; this method is known as HPLC-FLD [16]. Fluorescence is the process of a molecule’s electrons being raised to an excited state when they absorb light energy, followed by the electrons returning to a ground state and light being emitted. A fluorescence detector can capture the specific excitation and light emission wavelengths for a given molecule on the sample, whereas a UV detector would only capture light wavelengths. This can allow fluorescence detectors to be more selective than UV detectors, which may mean they can detect results when there are interferences in the chromatogram. As a result, an HPLC-FLD system can be more robust than a HPLC system with a UV detector.

#### 2.2.4. Laser-Induced Fluorescence Spectroscopy

More recently, the Laser-Induced Fluorescence Spectroscopy (LIFS) method has been used to detect the levels of aflatoxin in pistachios. Although, TLC and HPLC produce very accurate results, they are expensive, time-consuming, and involve destroying the sample in order to test it. They also cannot be implemented in an on-line manner, and due to their sample-based assessments, the entire batch a contaminated sample comes from needs to be destroyed, creating significant economic loss. Consequently, using optical methods for testing seems to have a lot of potential. They require minimal tampering of the pistachios before testing and so more product is able to be tested at a faster, cheaper rate [17].

The LIFS system works by inducing fluorescence on the pistachio electrons using a laser. This all takes place in a lighting chamber to eliminate contamination from external lighting. Then, the emissions are filtered and collected for each pistachio. It is a common practice to collect these results a few times for each pistachio and then average the results, in order to improve accuracy. To train the system, labelled samples of pistachios are used in three categories: uncontaminated, control, and contaminated. Control pistachios have a small amount of another substance (not aflatoxin) applied to a small amount of the surface area of the pistachio [17]. The contamination only appears on a small area of the pistachios surface and so a variety of methods are applied to the data to remove as much unnecessary information as possible, i.e., the data on the uncontaminated parts of the pistachio [17]. The first method applied is Principal Component Analysis (PCA), which is applied in order to find the principal components which can be used to build a discriminant model and to select the key wavelengths, important for developing a faster detection model [18].

Then, one of the following supervised learning methods is applied to distinguish whether pistachios are contaminated or not: Linear Discriminant Analysis (LDA), Logistic Regression Classification (LRC), or Support Vector Machine (SVM) [17]. LDA involves trying to minimise the interclass variance and maximise the distance between the means of the classes [19]. LRC involves defining a regression formula by which the data will be separated; the sigmoid/logistic function: $\sigma \left(x\right)=\frac{1}{1+e^{-x}}$ is commonly used [20]. SVM involves maximising the distance between a hyperplane (separating classes) and the closest data points in each of the classes [19]. In each method, there would be two classes: control and contaminated pistachios.

Researchers [17] compared the performance of the LDA and SVM approaches on LIFS data. Both approaches had very similar classification accuracy on the training data but the SVM model had a better classification accuracy on the validation data in each of the cases, with an equal or lower rate of false negatives on all of the data [18]. Qifang Wu, Huirong Xu, and Jun Xu compared the performance of SVM and LRC on LIFS data and found that the SVM model had higher classification accuracy for control, uncontaminated, and contaminated pistachio detection in each case [17]. Therefore, using an SVM model seems to be the best option for performing classification on the data from an LIFS system.

#### 2.2.5. Summary

Both TLC and HPLC are very invasive methods which require pistachios to be ground up and mixed with solvents to be tested. This means that many pistachios are being wasted for testing and can no longer be sold or consumed. If there was a method which did not involve tampering with the nuts themselves then this could save pistachio suppliers money as well as reduce their amount of waste.

The LIFS method allows analysis to take place on pistachios without destroying them; as a result there is less waste. It can also be incorporated in an on-line approach, allowing for more regular sampling and thus less product to be thrown away if contamination is identified. Therefore, this method is better for the economy and the environment. However, the samples do still need to be moved to a lighting chamber, where the testing takes place.

### 2.3. Hyperspectral Imaging

#### 2.3.1. What Is Hyperspectral Imaging?

Hyperspectral Imaging (HSI), also known as image spectroscopy, is a technique which involves photographing an object using many optical bands with a broad spectral range [21]. Its first applications were in the field of satellite and airborne imaging, where the term ‘Hyperspectral Imaging’ was coined [22]. Since then HSI has been used in numerous industries, including medical diagnosis, crime scene analysis, and food quality control [21].

**Figure 2:**
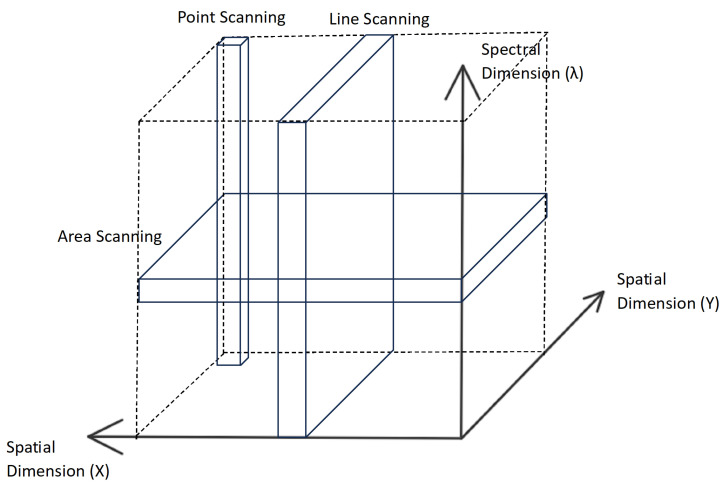
Point, line, and area scanning representation.

There are four main methods used to gather hyperspectral data: point scanning, line scanning, area scanning, and single shot systems, represented in Figure 2. In point scanning, the area under each pixel is scanned spatially and the spectral information of each point below the pixel is gathered. The scanner iterates along the X and Y spatial axes to scan every pixel of the sample. This process requires very precise hardware which is capable of moving by 1 pixel each time it scans a point. The process also takes a very long time due to the detail of its data [23].

In line scanning, the spatial and spectral information of a whole line of the sample is gathered at once. Then, the scanner iterates through each line of the sample. In both point and line scanning techniques, spatial data are collected in a logical order and build up as each point/line is scanned. However, spectral data are collected as a whole [23].

In area scanning, the opposite occurs, spectral data are collected in a logical order, building up, and spatial data are collected as a whole. Single-shot systems gather both the spatial and spectral information of the object in a single shot. Therefore, they do not require a scanning process to gather the information [23].

To capture hyperspectral (HS) image data, we used the benchtop HSI system from Resonon Hyperspectral Imaging Solutions, based in Bozeman, United States. This system includes a Pika XC2 HSI camera, an objective lens, a linear translation stage, a mounting tower, a halogen line light with a stabilised power supply, a calibration tile, and a system pre-loaded with Spectronon software. The Pika XC2 camera features a spectral range of 400–1000 nm, a spectral resolution of 1.9 nm, 447 spectral channels, 1600 spatial pixels, and a spectral bandwidth of 1.3 nm. Each pixel in the HS image contains a series of reflectance values across various spectral wavelengths, revealing the spectral signature of that pixel.

**Figure 3:**
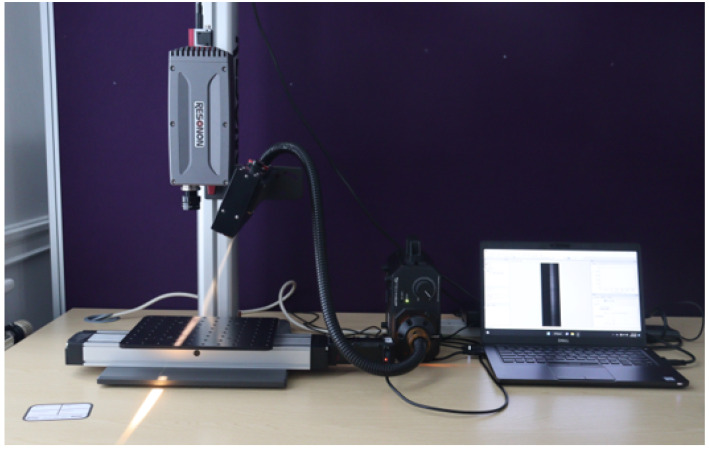
The camera setup used to gather the pistachio image dataset used for this paper. The camera uses the line scanning technique.

To set up the HS data acquisition system, the camera is mounted on the tower directly above the motorised linear translation stage. The lighting assembly is positioned and secured on the tower to illuminate the stage baseplate from above. A halogen line light provides stabilised broad-band illumination on the sample to be captured. To optimise the setup and improve data acquisition capabilities, the camera and lighting were carefully adjusted along the length of the tower. Figure 3 shows the HS image data acquisition setup.

To initiate data capturing, the camera was calibrated to ensure precise measurements. Throughout the data acquisition process, a consistent distance was maintained between the camera lens and the stage baseplate. During data acquisition, the linear translation stage moves, causing the tray of pistachios to be translated beneath the camera. The HS camera uses the push-broom technique for imaging, which involves scanning the object line-by-line using its inbuilt tunable filters or liquid crystal filters. By electronically adjusting these filters, the camera captures different spectral wavelengths of light. As the linear translation stage moves along the scanning direction, the camera sequentially captures HS information from different parts of the object. This process allows the construction of spectral intensity images for each wavelength, resulting in a comprehensive HS image set [24].

Hyperspectral data are represented by a hypercube, which is a 3D data structure. The dimensions are the two spatial domains (X and Y) and the spectral domain (λ), which can be seen in Figure 2. The data can be viewed in the form of an image at each λ, i.e., wavelength, or a spectrum at each spatial position (X,Y) [23].

#### 2.3.2. Hyperspectral Imaging in the Nut Industry

Hyperspectral Imaging has useful applications in the food industry for detecting the quality of foods. However, it requires expensive equipment and takes a long time to scan food samples; thus, so far, it has been most commonly used in research to identify which wavelengths are important for detection. The specific food industry can then use a multispectral imaging system, which captures data at these wavelengths; this is a much cheaper alternative to a hyperspectral imaging system [25].

There has been a variety of applications using HSI to detect specific areas, e.g., shell and/or contamination on a variety of different nuts, for example, distinguishing walnut shells from their meat. Zhu et al. [26] used fluorescence hyperspectral imagery in combination with Independent Component Analysis and a K-Nearest Neighbour classifer (ICA-kNN) in order to differentiate the parts of a walnut. Independent Component Analysis (ICA) selects the optimal wavelengths, then the K-Nearest Neighbours (kNN) classification is only applied to these optimal wavelengths. Their results showed that the ICA-kNN classifier using optimal wavelengths performed as well as the kNN classifier using all of the wavelengths, despite the ICA-kNN classifier being more efficient.

Jiang et al. [27] focused on the similar problem of distinguishing the shell and pulp of a black walnut using hyperspectral fluorescence imagery. However, instead of using an ICA-kNN approach, they used a Gaussian kernel-based support vector machine (SVM). They used the Gaussian kernel: $k\left(x,y\right)=\exp\left(-\frac{{∥x-y∥}^{2}}{2\sigma^{2}}\right),\sigma >0$, and found that the SVM with Gaussian kernel σ=0.1 had the best classification performance. The kernel was applied to map the hyperspectral image to a higher-dimensional space so that it was more linearly separable, to help make it easier to classify. Then, the performances of Principal Component Analysis (PCA), Fisher’s Distriminant Analysis (FDA), and Support Vector Machine (SVM) were compared using the kernal data. The SVM model had the best classification results.

Nakariyakul et al. [28] used hyperspectral imaging to classify almond nuts dependent on whether they were internally damaged or not. It is difficult to distinguish whether an almond is internally damaged by its external appearance. Hyperspectral images were taken of a sample of almond nuts. Then, the researchers reduced the amount of spectral bands analysed by eliminating one band if it was very similar to another band, starting with the most similar bands. The band ratios were then calculated and the list of the possible best two pairs of band ratios was narrowed down using the sequential forward selection (SFS) algorithm. Then, an SVM classifier with a Gaussian kernal was used to classify the almonds, trying out numerous different options of band ratios to find the ones which performed the best on the validation set. The model managed to achieve a correct classification rate of 90.16% on the training dataset and 93.33% on the validation dataset.

#### 2.3.3. Analysis of Hyperspectral Images of Pistachios

As well as the pistachio aflatoxin detection methods discussed previously, short-wave infrared hyperspectral images have also been used for detection. Bonifazi et al. [29] investigated various classification methods, applying them to short-wave infrared hyperspectral images. Using MatLab^®^ (R2023b, 2023), they began by testing out various pre-processing strategies and selecting the method that produced the best classification performance. They chose to try out and compare four commonly used classification methods: Partial Least Squares-Discriminant Analysis (PLS-DA), Principal Component Analysis with Discriminant Analysis (PCA-DA), Principal Component Analysis-based K-Nearest Neighbours (PCA-kNN), and Classification and Regression Tree (CART). The key concepts of these methods are described below.

PLS-DA consists of Partial Least Squares (PLS) regression used for dimensionality reduction using a dependent variable y, which contains the class information for the pistachios; y would be −1 for uncontaminated pistachios and 1 for contaminated pistachios [30]. This is then used to build a discriminant function using cross-validation, which should be able to assign a class to unknown data [29].

PCA-DA involves using PCA to reduce the number of variables to be used in discriminant analysis. The ideal number of principal components is found by using cross-validation [29]. Then, discriminant analysis is performed to identify characteristics specific to the distinct groups in order to classify the pistachios [31].

PCA-kNN uses PCA to reduce the amount of data used in the kNN algorithm by creating a score matrix which transforms the original hypercube into a reduced-dimensional space using the principal components [32]. The K-Nearest Nearest Neighbours (kNN) algorithm involves identifying the classes of the k-nearest neighbours of an unknown vector from the training data. The Euclidean distance is normally used to assess which vectors are nearest. The unknown vector is then classified by the most common class of its k-nearest neighbours [33].

CART analysis is a tree-building technique, which uses binary recursive partitioning. This means that a node of the tree can only have two child nodes. CART splits the data up into a tree, labelling resulting nodes with a class until it stops when it has achieved a maximal tree. A maximal tree is likely to overfit the training data and so pruning occurs to create numerous simpler trees from the maximal tree with lower complexity. The optimal tree is then picked based on its performance on the validation data. This tree is used to classify the data [34].

Bonifazi et al. [29] conducted investigations which showed that the PCA-kNN model produced the best predictive model, although all of the models performed relatively well in classification. Their models were able to classify the stone, twig, shell, and husk of the pistachio, and which parts of the pistachio were edible/inedible.

#### 2.3.4. Summary and Insights

The previous methods used for pistachio aflatoxin hyperspectral analysis are all supervised learning techniques trained on pixel-by-pixel breakdowns of the areas of the pistachios that contain aflatoxin and those that do not. These methods then take in a hyperspectral image of a sample of pistachios and use the trained model to classify it. However, the dataset in this paper only labels the overall level of aflatoxin in the pistachio sample imaged, not a pixel-by-pixel breakdown of where the contamination is. Therefore, the paper explores the application of an unsupervised learning technique, K-Means Clustering, and supervised learning techniques that label the image as a whole, to the hyperspectral images of pistachios. The pistachio suppliers only need to judge the level of the sample; they do not require a pixel-by-pixel breakdown of the image. Consequently, it would be simpler and more efficient to build a solution that classifies the image into the correct level of aflatoxin without outputting a full pixel-by-pixel breakdown.

## 3. Materials and Methods

This section discusses the machine learning techniques which have been used to classify and evaluate the hyperspectral images of pistachios. These techniques are used to classify the level of aflatoxin in the imaged sample of pistachio nuts or to create clusters within the image which could indicate areas of aflatoxin.

### 3.1. Residual Network (ResNet)

Convolutional Neural Networks (CNNs) are made up of neurons which through learning optimise themselves. The neurons receive an input, raw image pixel values in the case of image classification, and apply operations to the input. The output of these operations is then passed to the next layer. In the final layer of the CNN, there are loss functions which are applied in order to classify the image [35].

**Figure 4:**
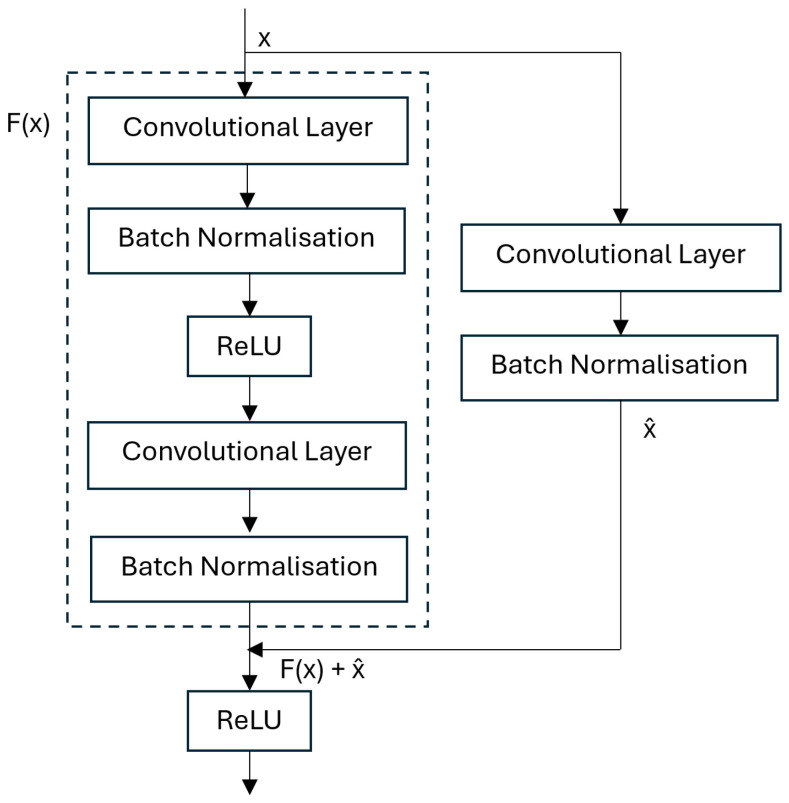
Residual Network Block implemented in the Residual Network Architecture.

When layers are added to CNNs, they experience a degradation of training accuracy. Residual Networks (ResNets) solve this problem by introducing an identity mapping [36]. The result of the operation f(x) as well as the input into the operations *x* are passed into the activation function as f(x)+x, as seen in Figure 4.

This helps to solve the degradation problem because accuracy does not saturate as the network gets deeper, due to the identity mapping bypass connection. This bypass connection also helps to reduce the vanishing gradient problem because the gradients can bypass the operations. Consequently, ResNet architectures can be very deep, allowing them to produce state-of-the-art results, outperforming the traditional CNN. They rose to popularity after winning the ILSVRC 2015 classification competition [36,37]. Zhong et al. [38] compared the performance of Deep Residual Networks (ResNets) to Convolutional Neural Networks (CNNs) for pixel-wise classification of hyperspectral images. They conducted many experiments, varying the number of layers in the network and the size of the training dataset, and in 95% of cases, the ResNet had a higher accuracy than the CNN. An et al. [37] used a ResNet architecture to successfully classify images of nuts, using transfer learning after training the model on the ImageNet dataset.

### 3.2. Variational Autoencoder

An autoencoder is an unsupervised learning method normally consisting of three layers: an input, bottleneck, and output layer. Firstly, the inputs *X* get encoded into feature-extracted representations *Z*, by Z=f(WX+b) in the bottleneck layer, where *W* represents the vector of weights, which are learned during training, and *b* is the bias. Then, *Z* is decoded into $\hat{X}$, which is of the same form as *X*. The loss is calculated between *X* and $\hat{X}$ and the error is then backpropogated through the network [39].

A Variational Autoencoder (VAE) uses an autoencoder to generate data by assuming that the input data *x* are produced using $p_{\theta }\left(z\right)$, by a true prior latent distribution z, where θ are the model parameters. Data are generated by sampling x using the true conditional $p_{\theta }\left(x|z^{i}\right)$, and estimating the true parameters of θ. This true conditional is approximated by $q_{\varphi }\left(z|x\right)$ so that it is not intractable and the likelihood of the training data can be maximised. Using the reparameterisation trick, the likelihood can be maximised by maximising a reconstruction term and minisiming the KL divergence ($KL(q_{\varphi }\left(z|x^{i}\right)||p_{\theta }\left(z\right))$) [39]. Training of the VAE attempts to maximise the following term to find the optimal θ and φ:

(1) $$\max_{\theta ,\varphi }\sum (E_{z}\left[\log p_{\theta }\left(x^{i}|z\right)\right]-KL(q_{\varphi }\left(z|x^{i}\right)\left|\right|p_{\theta }\left(z\right))$$

Bergamin et al. [40] discussed various ways in which VAEs could be used for anomaly detection. Anomaly detection systems have a variety of applications, including quality control. A VAE could be used to detect aflatoxin as the anomaly within images of pistachios.

#### t-Distributed Stochastic Neighbor Embedding

t-Distributed Stochastic Neighbor Embedding (t-SNE) is a technique which allows high-dimensional data to be visualised on a two- or three-dimensional map. This is performed by using a Student’s t-distribution to compare the similarity of two points in a low-dimensional space. t-SNE utilises a heavy-tailed distribution to reduce crowding within the lower-dimensional space [41]. It can be used to reduce the dimensionality of the encoded *Z* of a VAE to allow the distribution of the encoded input data points to be observed [42].

### 3.3. Deep Convolutional Generative Adversarial Network

A Deep Convolutional Generative Adversarial Network (DCGAN) consists of a discriminator and a generator, each of which have their own neural network. The generator network G uses transposed convolutional layers to generate images from d-dimensional vectors. The discriminator network D determines whether the image passed into it is a real image or an image generated by the generator. The learning process consists of two parts: training the discriminator network using the input dataset and the generator network generating an image from an arbitrary vector [43].

The discriminator network is then trained on the generated image. These steps are repeated in the hope of the generator producing images which are gradually harder for the discriminator to decipher from the original dataset [43]. This is represented as:

(2) $$\min_{G}\max_{D}V\left(D,G\right)=E_{x∼p_{data}\left(x\right)}\left[\log D(x)\right]+E_{z∼p_{z}\left(z\right)}\left[\log\left(1-D(G(z))\right)\right]$$

where *x* represents the original image from the dataset, $p_{data}\left(x\right)$ is the likelihood distribution of *x*, *z* is a d-dimensional vector of random numbers, and $p_{z}\left(z\right)$ is the likelihood distribution of *z* [43]. D(x) is the likelihood the image is not generated. The term 1−D(G(z)) is the likelihood the generated input is a generated image; the generator is trying to minimise this value. Meanwhile, the discriminator is trying to maximise its accuracy, leading to the generator producing better images to fool the discriminator [43].

Tan et al. [44] developed a DCGAN model to detect pesticide residues from hyperspectral images of Hami melon. The hyperspectral images were fed into a DCGAN model which outputted generated spectral data. These data were then classified into which pesticide the melon contained or no contamination. The classifier was able to achieve an accuracy of 82.5%, indicating that with a larger dataset, it would be feasible to build an accurate classifier using a DCGAN model.

### 3.4. Dimensionality Reduction with K-Means Clustering

Dimensionality Reduction with K-Means Clustering is an unsupervised approach to classifying a hyperspectral image into clusters. Due to the size of hyperspectral images, it is important to perform Dimensionality Reduction, in order to reduce the image’s dimensionality before performing the K-Means algorithm, otherwise the algorithm will take an extremely long time to run. The time complexity of the K-Means algorithm is O(TKn), where *T* is the number of iterations, *K* is the number of clusters, and *n* is the number of input patterns (size of the hyperspectral image). However, the effective time complexity is $O(n^{2})$ because *T* is approximately proportional to *n* [45]. Consequently, reducing the dimensions (the size of *n*) significantly reduces the time complexity of the algorithm.

The K-Means algorithm involves selecting k initial centroids and then determining the nearest centroid to each data point. The data points for each centroid are then grouped together into clusters, and the centre point of each cluster is calculated to find the new centroid. This process is repeated with the new centroids until no data points change clusters or the maximum number of iterations T is reached [46]. The number of clusters k needs to be specified in advance of running the algorithm.

Zhu et al. [47] used K-Means Clustering after PCA (dimensionality reduction) to cluster hyperspectral images of peanut kernels in order to identify aflatoxin B1. The K-Means Clustering method achieved a validation accuracy of 96.77%, indicating that it is possible to identify aflatoxin B1 in peanuts using K-Means Clustering. The study used a dataset with 12,374 images in total.

### 3.5. Evaluation Metrics

The following evaluation metrics were used to assess the performance of the classifiers. Firstly, a confusion matrix was produced for each classifier; this contained the number of false positives (FP), false negatives (FN), true positives (TP), and true negatives (TN) the model predicts. The performance of the models was evaluated using a confusion matrix. The results for accuracy, precision, recall, and F1-score were calculated using the following equations and tabulated in Table 1:

(3) $$\mathrm{Accuracy}=\frac{TP+TN}{TP+TN+FP+FN}$$

(4) $$\mathrm{Precision}=\frac{TP}{TP+FP}$$

(5) $$\mathrm{Recall}=\frac{TP}{TP+FN}$$

(6) $$F1-\mathrm{Score}=2\times \frac{\mathrm{Precision}\times \mathrm{Recall}}{\mathrm{Precision}+\mathrm{Recall}}$$

Accuracy evaluates the overall correctness of the model, although it is important to also use other metrics, especially when the dataset is not balanced. This is because the model may just pick the most common class. A high training and test accuracy indicates that the model is not overfitting to the training data.

The cost of a model indicating that the pistachios are okay to consume when they are not (false negative) is very high because it could lead to consumers having liver cancer [2]. Therefore, it is important to evaluate the model’s recall. A high recall indicates there are low numbers of false negatives. The precision evaluates the amount of false positives within the model. A high precision indicates a low number of false positives, leading to less pistachios being identified as contaminated when they are not. A low precision could lead to unnecessary waste. The F1-score combines these metrics to create one score which can be used in comparison.

All of the classifiers developed were evaluated on these metrics and the model with the highest accuracy was chosen as the most effective model to be used on this specific pistachio hyperspectral image dataset. Similar papers on other types of food have managed to achieve accuracies around 90% on the highest performing models [25,26,27,28,29]. However, due to the size of the dataset, creating a model which had training and test data accuracy above 80% with a high precision and recall would indicate that with a larger dataset the same model may achieve higher accuracy and be considered to replace existing detection methods.

## 4. Design and Implementation

One of the main aims of the project was to test the feasibility of classifying the aflatoxin level of pistachios using only a single hyperspectral wavelength. To test this, three machine learning techniques, Residual Networks, Variational Autoencoders, and Deep Convolutional Generative Adversarial Networks, were applied to the images from an individual wavelength. An unsupervised method Dimensionality Reduction with K-Means Clustering was also applied to the whole hyperspectral image to compare to these classifiers. None of these methods had been applied to hyperspectral images of pistachios which were not labelled pixel-wise before.

Their ability to learn complicated characteristics from a single wavelength allows them to extract important patterns from hyperspectral images (HSI), improving classification performance. When labelled data are limited, unsupervised classification is made achievable by combining Dimensionality Reduction with K-Means Clustering. Also, GANs and VAE can help in covering up data shortage by creating synthetic data points using the existing dataset. Applying these techniques to HSI classification of pistachios could improve aflatoxin detection, offering a novel, data-driven approach for food safety and quality control.

### 4.1. Data Processing

#### 4.1.1. Data Format

The dataset of pistachio hyperspectral images used for this paper was taken and provided by Leeds Beckett University. The dataset featured only 300 hyperspectral images of groups of pistachios with corresponding text files, each containing the details of its hyperspectral image. Each hyperspectral image and corresponding text file were labelled into one of three classes (low, medium, high) based on the their aflatoxin level. There were 100 images for each class of infection: Less Than 8 μg/kg (low), Greater Than 160 μg/kg (medium), and Greater Than 300 μg/kg (high). The data were not labelled to show which pixels contain aflatoxin (pixel-wise); they were only labelled to indicate the overall aflatoxin level of the pistachios within each image. Therefore, any image segmentation to indicate parts containing aflatoxin required unsupervised learning, but a classifier could use the label of the overall image.

The hyperspectral images from the Less Than 8 ppn (μg/kg) and Greater Than 300 ppn (μg/kg) levels included pistachios with shells, and the images from the Greater Than 160 ppn (μg/kg) level included pistachios without shells. Each of the hyperspectral images were made up of 462 wavelengths, ranging from 386.88 nm to 1003.6 nm. Each hyperspectral image was approximately 3.5 GB, and the overall dataset was approximately 1.1 TB. Therefore, these large files took a long time to process and required a very considerable amount of storage. The images did not all have the same dimensions, they all had 462 wavelengths and a width of 1600 pixels but the height of the images ranged from 1800 to 2400 pixels. The images were all taken using the same hyperspectral camera, base and bowl, although the bowl sometimes moved locations between each image. The bowl contained a singular layer of pistachios scattered in a random assortment. Pre-processing was introduced, i.e., cropping and rotating, to help to prevent overfitting to training data of a particular assortment of pistachios or background.

**Figure 5:**
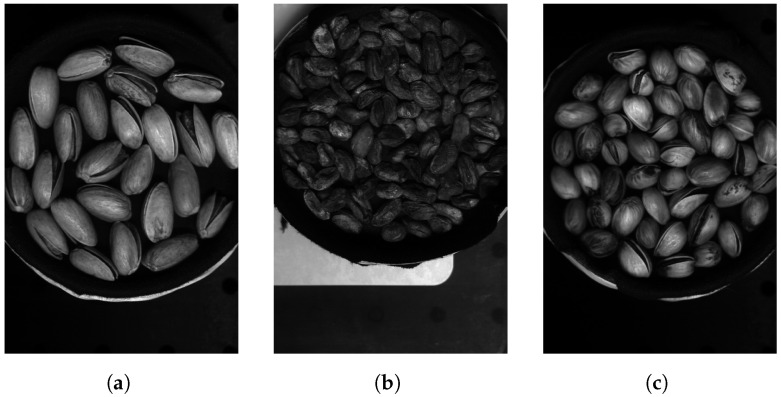
Representation of the 453.8 nm wavelength at the 3 different aflatoxin levels: (**a**) Less Than 8 μg/kg, (**b**) Greater Than 160 μg/kg, and (**c**) Greater Than 300 μg/kg.

**Figure 6:**
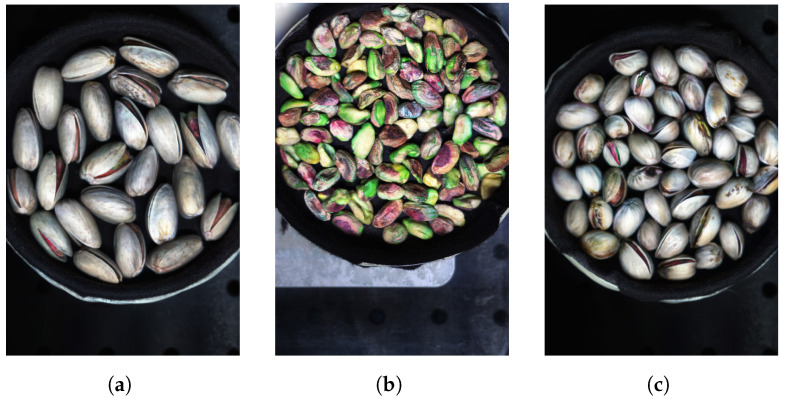
RGB representation of hyperspectral images at the 3 different aflatoxin levels: (**a**) Less Than 8 μg/kg, (**b**) Greater Than 160 μg/kg, and (**c**) Greater Than 300 μg/kg.

**Figure 7:**
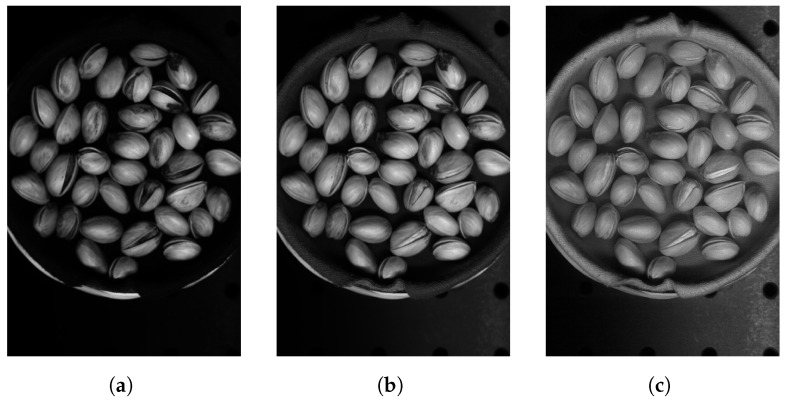
Three different bands of the same hyperspectral image of pistachios from the Greater Than 300 μg/kg level: (**a**) Band 151 (534 nm), (**b**) Band 241 (628 nm), and (**c**) Band 461 (1003 nm).

The overall hyperspectral image could be processed to view each individual band corresponding to a wavelength of the hyperspectral image. The hyperspectral image could also be compressed into an RGB representation, as seen in Figure 5, Figure 6 and Figure 7.

#### 4.1.2. Breakdown into Individual Wavelengths

Hyperspectral cameras, though costly, capture images across numerous spectral bands. Aflatoxins, toxic substances produced by mould, can be detected within specific spectral bands. By identifying the precise spectral bands necessary for accurate aflatoxin detection, it may be feasible to use a more affordable multispectral camera. This camera would target only the essential spectral bands required for identifying aflatoxins on pistachio nuts, offering a cost-effective solution for suppliers to test aflatoxin levels in their products. The hyperspectral images were analysed to find the important wavelengths which could identify the aflatoxin level of the pistachios pictured. Then, classifiers were created using only the images from an individual wavelength to test whether it was possible to classify a sample using just the information from those wavelengths.

The first step was to break the hyperspectral images down into images of each individual wavelength. There were 300 hyperspectral images and each had 462 wavelengths, resulting in 138,600 wavelength images. The breakdown of each hyperspectral image took a long time due to their size. The hyperspectral images were processed into individual wavelengths using MatLab and the ‘hypercube’ function in the Image Processing Toolbox Hyperspectral Imaging Library add on. This add on was not installed on the university lab machines and could not be added. Therefore, it was not possible to use Condor to automate and process the hyperspectral images in parallel. This meant that the processing of each of the 300 hyperspectral images into individual wavelength images had to be manually started. A for loop was used to reduce the amount of times the processing of images had to be manually started.

#### 4.1.3. Key Wavelength Analysis

In order to identify the range of wavelengths which show the aflatoxin within the pistachios, the principal wavelengths of each hyperspectral image needed to be analysed. This was performed using MatLab and the ‘selectbands’ function in the Image Processing Toolbox Hyperspectral Imaging Library.

The ‘selectbands’ function that was used begins by removing the low signal-to-noise (SNR) bands, which are generally distinctive but not informative. This is performed by removing bands that have low spectral correlation coefficients with their adjacent bands. The noise component within each band varies, which might falsely indicate that the band is very informative. Therefore, data-whitening is performed, using an eigen decomposition of the data covariance matrix, to moderate the noise.

After pre-processing, the function finds the desired amount of principal bands by first finding the pair of bands with the highest dissimilarity. This is performed by selecting a random band $W_{1}$ and projecting all of the other bands onto the orthogonal subspace of that band. The band with the maximal projection $W_{2}$ is the most dissimilar to the original band. The process is repeated, projecting the rest of the bands on the orthogonal subspace of the band with the previous maximum projection $W_{2}$, to find the new maximum $W_{3}$. $W_{1}$ and $W_{2}$ are confirmed to be the pair with the most dissimilarity if $W_{1}$ = $W_{3}$. If $W_{1}$ ≠ $W_{3}$, the process is repeated until $W_{i-1}$ = $W_{i+1}$, where $W_{i-1}$ and $W_{i}$ are selected as the most distinct pair of bands [48].

This pair then forms the subset of the most informative bands; the subset is expanded by finding the band with the highest dissimilarity from the bands in the subset. The band with the highest dissimilarity from the subset is found using the orthogonal space projection (OSP) method. OSP begins by constructing an orthogonal subspace of the subset of bands as: $P=Z{\left(Z^{T}Z\right)}^{-1}Z^{T}$, where I is an N×N identity matrix and Z is an N×M matrix, where each column corresponds to the pixels of a band in the subset and *M* is the amount of bands within the subset. The projection $y_{0}=P^{T}y$ is calculated for each remaining band, where y is the pixels of that band. The highest $∥y_{0}∥$ indicates the band with the maximal dissimilarity from the subset. This band is then added to the subset and the process is repeated until the subset is of the desired size [48].

The five most informative bands were computed for each of the 300 hyperspectral images. Due to the reasons discussed in Section 4.1.2, Condor could not be used for this process. Therefore, the code was run manually for each hyperspectral image. It took approximately 5 min for the key wavelengths of each image to be identified, consequently, approximately 25 h for all of the images to be analysed.

The Greater Than 160 ppn images contained hyperspectral images of pistachios without shells, whereas both of the other two levels’ datasets contained hyperspectral images of pistachios with shells. Consequently, the differences in important bands between these two types of hyperspectral images could be due to the information from pistachio images without shells appearing more prominent in different bands compared to images of pistachios with shells. The classifiers might be able to identify the Greater Than 160 ppn level by learning features unique to pistachios without shells. The main difference between the other two levels’ appearances should be the presence of aflatoxin. Therefore, it was more important for the classifiers to distinguish between these two levels.

**Figure 8:**
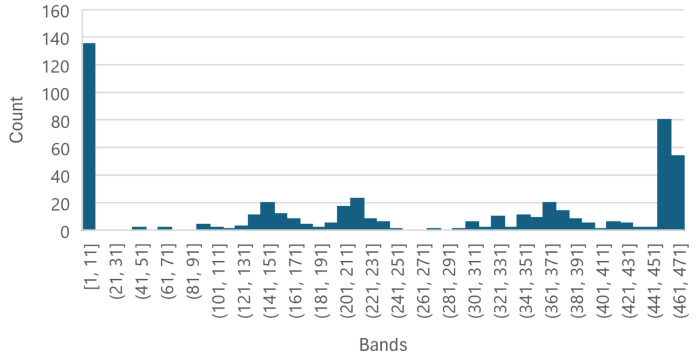
Histogram of the five most informative bands for each Less Than 8 ppn hyperspectral image using the ‘selectbands’ function as described above. There are five peaks at [1, 11], [141, 151], [211, 221], [361, 371], and [451, 462].

**Figure 9:**
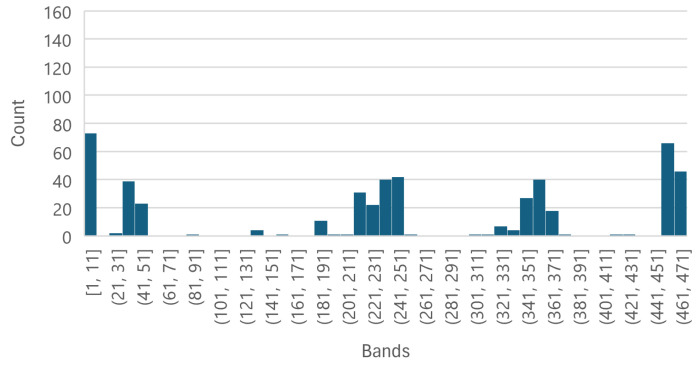
Histogram of the five most informative bands for each Greater Than 160 ppn hyperspectral image using the ‘selectbands’ function as described above. There are five areas which contain the majority of the bands selected [1, 11], [21, 51], [211, 251], [341, 371], and [451, 462].

**Figure 10:**
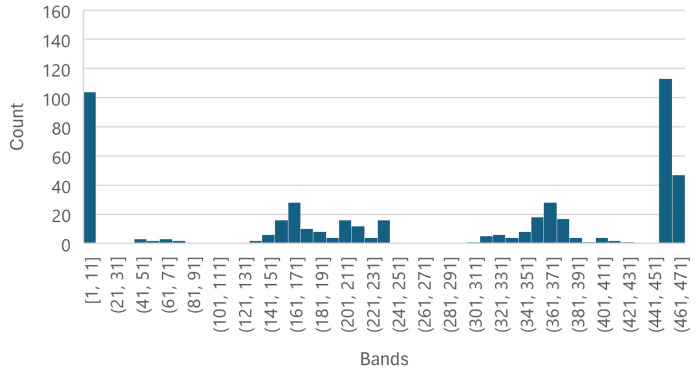
Histogram of the five most informative bands for each Greater Than 300 ppn Hyperspectral Image using the ‘selectbands’ function as described above. There are four peaks on the histogram at [1, 11], [151, 171], [361, 371], [451, 462].

Each of the Figure 8, Figure 9 and Figure 10 shows that the five most important bands of each level have a peak at [1, 11], leading to the selection of band 11, corresponding to wavelength 399.98 nm, to be used as an individual wavelength for the classifiers. Similarly, the ranges [361, 371] and [451, 462] have a peak in each Figure 8, Figure 9 and Figure 10 as well; thus, bands 361 and 461, corresponding to wavelengths 866.21 nm and 1002.23 nm, respectively, were selected for the classifiers.

**Figure 11:**
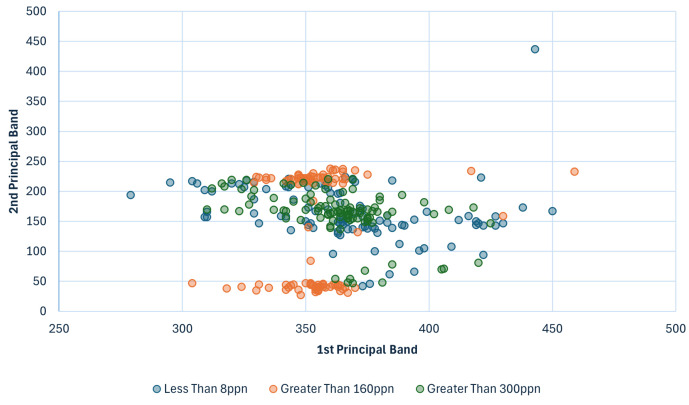
Pairs of the most informative bands for the hyperspectral images from each level: Less Than 8 ppn, shown in blue circles, Greater Than 160 ppn, shown in orange circles and Greater Than 300 ppn, shown in green circles. At the Greater Than 160 ppn level, there are two clear dense clusters, centred approximately around points (355, 45) and (350, 225). The Less Than 8 ppn and Greater Than 300 ppn distribution of points are much more comparable, their main clusters seem to both be centred approximately at the point (365, 150) although the standard deviation of the points for both levels is much greater than for the Greater Than 160 ppn level. Ignoring outliers, the 1st Principal Band ranges from band 300 to 425, and the 2nd Principal Band ranges from band 25 to 241.

The Less Than 8 ppn graph (Figure 8) has a peak around [141, 151] and the Greater Than 300 ppn graph (Figure 10) has a peak around [151, 171], so band 151, corresponding to wavelength 584.64 nm, was selected to be used for the classifiers. Figure 9 has a peak around [211, 251] and Figure 11 has a cluster for the Greater Than 160 ppn dataset centred at a second most principal wavelength between 200 and 250, resulting in the final wavelength for analysis being selected at band 241, corresponding to wavelength 704.64 nm.

Each of the datasets from these wavelengths was used to evaluate the performance of the classifiers to gain an understanding of the important wavelength region to guide further research.

## 5. Results

### 5.1. Dimensionality Reduction with K-Means Clustering

#### 5.1.1. Dimensionality Reduction

Before applying the K-Means Clustering algorithm, the dimensionality of the hyperspectral images needed to be reduced. This was especially important considering the K-Means algorithm has an effective time complexity of $O(n^{2})$, where n is the dimensions of the input image. The n of the whole hyperspectral image was 2400 × 1600 × 462 = 1,774,080,000. Dimensionality reduction was performed using MatLab and the ‘selectbands’ function in the Image Processing Toolbox Hyperspectral Imaging Library, as described in Section 4.1.3. The number of bands was reduced from 462 to the 10 most important bands, which contain the majority of the information. This made n 46.2 times smaller and the time complexity 2134.44 times smaller. There was a trade-off between the number of wavelengths to retain and the time complexity of the K-Means algorithm. The more bands retained, the more information passed into the clustering algorithm to be used to assign clusters. However, the more bands, the longer both algorithms took to run. Considering that the K-Means algorithm had a quadratic effective time complexity, the extra bands would have increased the time that the algorithm took to run drastically. The entire hyperspectral image took approximately 10 h to run for 10 clusters with only 10 iterations, and the hyperspectral image after Dimensionality Reduction with 10 bands for 10 clusters took approximately 1.5 h to run for 10 iterations. The ‘select-band’ function took approximately 6 min to run for each hyperspectral image.

#### 5.1.2. K-Means Clustering

The images contained the bowl and base of the hyperspectral camera, which varied between the images, depending on the position of the bowl. In order to stop the background from affecting the clustering results, before passing images into the clustering algorithm, the Less Than 8 ppn images were cropped to be half of the original dimensions of the input image (1200 × 800 × 10) and the Greater Than 300 ppn images were cropped to (900 × 600 × 10). This also helped to reduce the effective time complexity of the algorithm by 16 and 49 times, respectively. The algorithm now only took approximately 5 and 2 min, respectively, to run through to completion (no iteration limit). The Greater Than 300 ppn images were cropped more than the Less Than 8 ppn images for visual experiments because the pistachios within the Greater Than 300 ppn images appeared smaller; hence, cropping the images differently allowed the pistachios to appear the same size.

The K-Means algorithm requires that k, the number of clusters, be selected before the algorithm begins to run. This number was important because if the number was too low then important features could be missed off, but if the number was too high, there may be redundant segments created. The number of clusters also impacted the time complexity of the algorithm, so it was important to balance having enough clusters to capture the important information (aflatoxin) but not having too many so that the algorithm took a long to run and there were excessive clusters.

**Figure 12:**
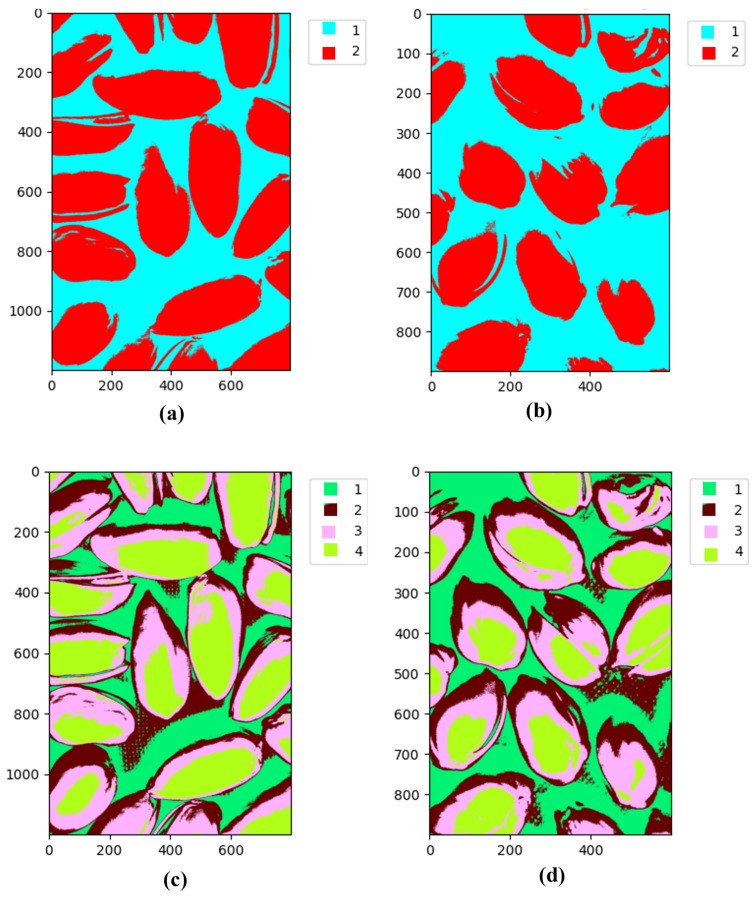
K-Means clustering results following dimensionality reduction: (**a**) for a less than 8 ppn hyperspectral image with 2 clusters; (**b**) for a greater than 300 ppn hyperspectral image with 2 clusters; (**c**) for a less than 8 ppn hyperspectral image with 4 clusters; and (**d**) for a greater than 300 ppn hyperspectral image with 4 clusters.

The final algorithm was applied to images from the Less Than 8 ppn and Greater Than 300 ppn levels which contained pistachios with shells. The algorithm was implemented using Python’s Spectral module. To decide the number of clusters to use, the clustering results for an image from each of these levels for k = 2 to 15 were analysed. Figure 12a–d clearly show that when only 2 and 4 groups were used, there were too few clusters to distinguish important details between the images. There are no clear visual distinctions between the two levels, and the clusters are only able to represent the main details of the images, e.g., background and pistachio shell.

**Figure 13:**
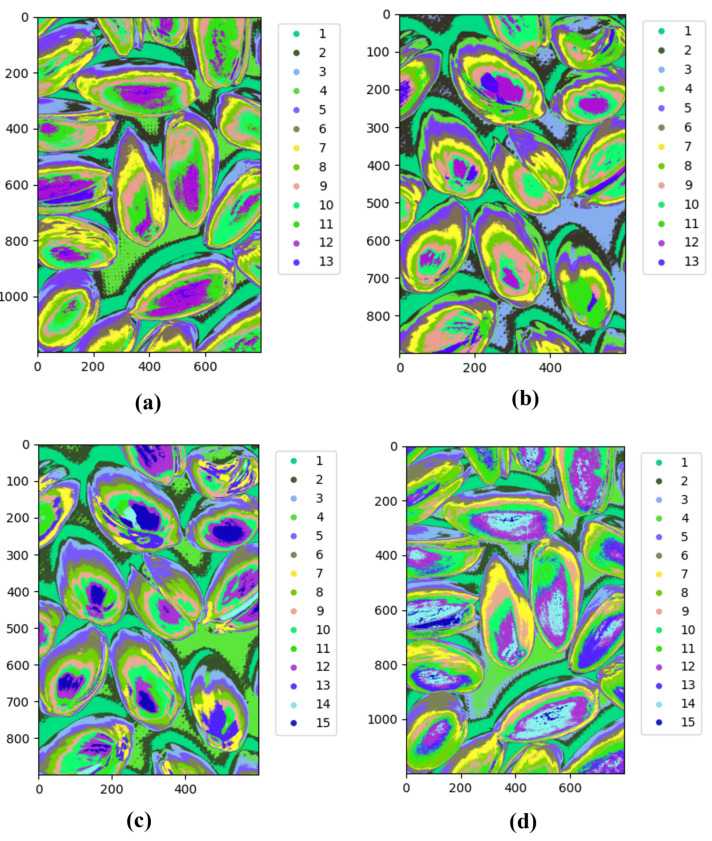
K-Means clustering results following dimensionality reduction with various clusters: (**a**) less than 8 ppn hyperspectral image with 13 clusters; (**b**) greater than 300 ppn hyperspectral image with 13 clusters; (**c**) less than 8 ppn hyperspectral image with 15 clusters; and (**d**) greater than 300 ppn hyperspectral image with 15 clusters.

When 13 and 15 clusters were used, as shown in Figure 13a–d, it is easy to visually observe that for the Less Than 8 ppn image, unlike lower values of k, the clusters are much less well defined. Instead of being areas of block colours, there is much more overlap between clusters, e.g., an area that has some pixels of both clusters. This possibly indicated the presence of redundant clusters because the algorithm was struggling to distinguish which cluster a point belonged to. Here, k = 10 was selected for the clustering algorithm because the clusters were better defined, which indicated that there were no redundant clusters on the pistachios, but there were enough clusters to capture more information than just the background and the pistachio shells.

#### 5.1.3. Visual Experiment

**Figure 14:**
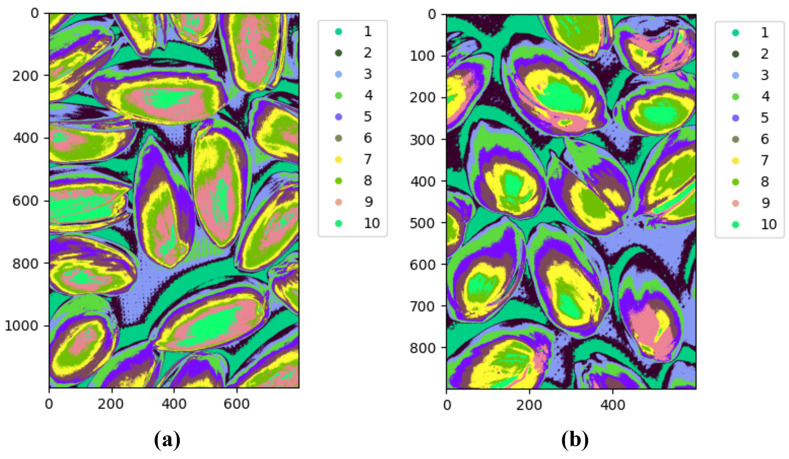
K-Means clustering results following dimensionality reduction with various clusters: (**a**) less than 8 ppn hyperspectral image with 10 clusters; (**b**) greater than 300 ppn hyperspectral image with 10 clusters.

Once deciding on using 10 clusters, the K-Means Clustering algorithm was run on 10 random hyperspectral images which had undergone Dimensionality Reduction: 5 from the Less Than 8 ppn level and 5 from the Greater Than 300 ppn level, using the same 10 colors for each of the images’ clusters. Then, an experiment was designed to evaluate whether people could see obvious visual differences between the images. The 10 images were used to create an image-matching exercise in which people had to assign five to one group and the five they had not selected were assumed to be the other group. They also had to enter their reasons for classifying the images. The experiment was conducted using Survey Monkey. Figure 14a,b show two of the images that were used in the sorting exercise.

In the image-matching experiment, 38 participants participated, but only 32 chose five pistachios and correctly answered the questions. The exercise’s lowest possible score was 60% because a group would be the other level if they were given zero of one of the level’s images. If someone assigned only one of a level’s images to their group, then their group had four of the other level, so they scored 80%. Finally, if someone assigned two of a levels’ images to their group, then their group contained three of the other level’s images, so they scored 60%. A total of 66.667% of participants only scored 60% on the exercise, 9.375% of participants scored 80%, and 15.625% scored 100%.

The experts who answered perfectly had reasons including the apparent shape of the pistachios and the presence of bright green within the images. These reasons did not necessarily relate to the presence of aflatoxin within the images and were not obvious to the other participants. Therefore, the experiment concluded that it was not possible for humans to visualise the difference between the different levels of pistachios following Dimensionality Reduction with K-Means Clustering.

#### 5.1.4. Pixel-by-Pixel Analysis

Following the conclusions of the visual experiment, the counts of each color within each of the pistachio images were investigated to assess whether there were any obvious differences between the distributions of colors within the two levels of contamination.

**Figure 15:**
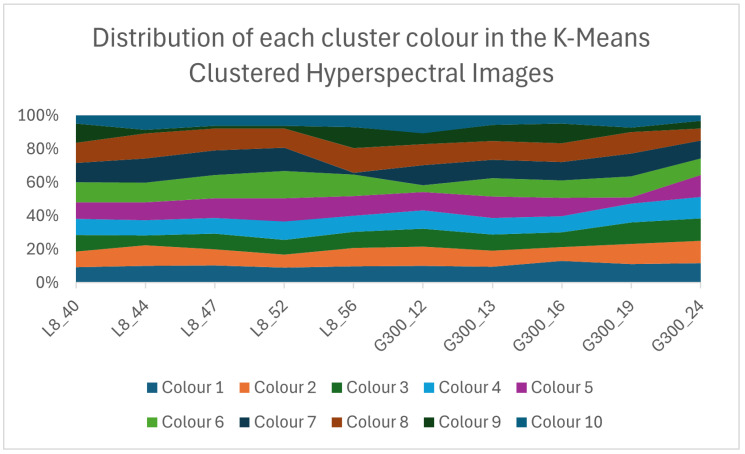
Distribution of colours within each K-Means Clustering of the reduced hyperspectral images after Dimensionality Reduction, using 10 clusters.

Figure 15 shows no drastic differences in the color distribution within the images at the two different levels. From initial observations, it appeared that there may be a higher percentage of color 6 within the Less Than 8 ppn images than in the Greater Than 300 ppn images.

**Figure 16:**
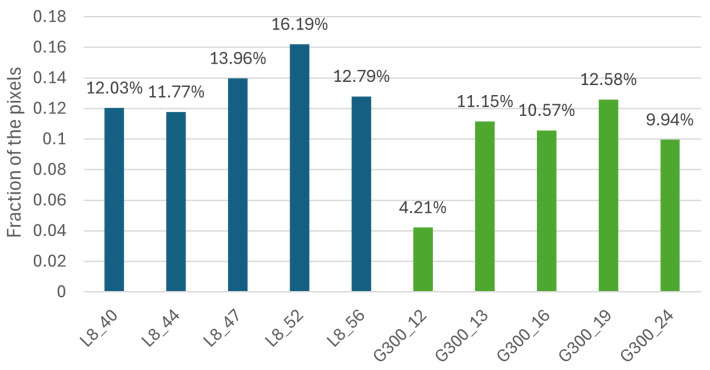
Percentage of colour 6 pixels compared to the overall pixel count for each K-Means Clustered image.

Figure 16 shows that apart from the 19th Greater Than 300 ppn cluster image, all Greater Than 300 ppn images had a lower percentage of color 6 than the Less Than 8 ppn images. For the 10 samples, there was a 90% classification accuracy when the percentage of colour 6 was greater than or equal to any percentage between 11.16% and 11.77%. This method of classification was evaluated on 22 more images. This 90% accuracy was a higher accuracy than would have been possible when selecting a value for any other colours’ percentages to classify the images.

**Figure 17:**
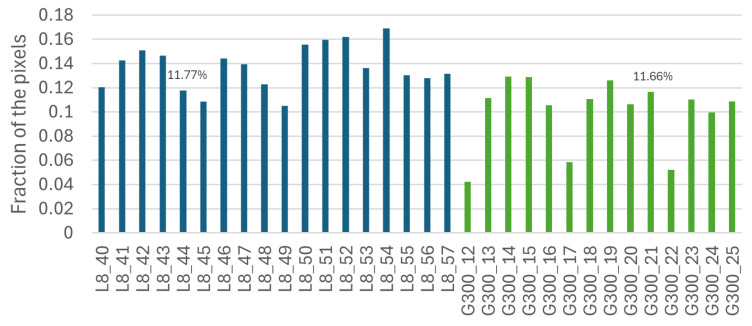
Fraction of colour 6 pixels compared to the overall pixel count for the extended K-Means Clustered Images.

The extended model had the highest classification accuracy when using a percentage between the value for images L8_44 and G300_21; so, a percentage between 11.77% and 11.66%, e.g., using 11.7%. The classifier was evaluated using metrics described in Section 3.5. To begin, a confusion matrix was developed based on the results from Figure 17 using 11.7%, as seen in Table 2.

#### 5.1.5. Follow-Up Experiment

Now that a quantifiable difference between the two types of image had been identified, the survey was conducted again, but this time, it guided the participants to focus on the quantity of colour 6 within the images. The experiment was conducted to discover if the participants were now able to visually distinguish between the clustered images with this information.

A camera would be implemented within a production line, and after the image of a sample of pistachios was taken and reduced in size, it would be passed through the K-Means Clustering algorithm. If the participants in the experiment were able to visually distinguish the images, then it was likely that a worker on the production line would be able to do so as well. Counting the number of pixels of colour 6 added time complexity to the overall function with a time complexity of O(wh), where *w* is the width of the image and *h* is the height of the image. Although this is a very low time complexity, when hundreds of thousands of pistachios are processed a day, this additional time adds up. However, this is still likely to be quicker than a worker visually assessing the amount of colour 6, which is also subjective.

The new experiment was the same as the previous experiment except the description guided the users to use the amount of colour 6, which was the brown colour within the images, to classify them. The false positive image G300_19, which was in the original experiment, was removed from the new experiment. Participants were also asked to say whether they had selected the five images which they believed had the most or least colour 6 so that the experiment did not just have three different scores.

A total of 13 people answered the new survey, 15.38% scored 100% (perfect classification), 23.08% scored 80%, 38.46% scored 60%, 15.38% scored 40%, and 7.69% scored 20%. No one scored 0% and the average score was 64.62%. This indicated that even with knowing the method to use to classify the images and the number of images at each toxicity level, humans could not classify the images as accurately as the counting computation. This experiment was easier for the participants than it would be on a production line because they knew that five belonged to one class and five to another. They could select the five with the most or least colour 6. In reality, the workers would not know how many of the pistachio samples were contaminated. Therefore, it was concluded from the experiments that it was not possible to visually use the K-Means Clustering maps to assess the level of aflatoxin within pistachios. However, the counting method using colour 6 had a high accuracy and F1-score, suggesting that it could be possible to develop a method by counting the pixels of specific colours to determine the aflatoxin level.

### 5.2. Residual Network (ResNet)

The purpose of the ResNet classifiers was to discover if it was possible to classify the aflatoxin level from only an individual wavelength and to identify which of the five wavelengths identified in Section 4.1.3 had the highest accuracy. This would indicate that this wavelength might be the best choice for a multispectral camera.

#### 5.2.1. Data Pre-Processing

Individual wavelength images were passed into the ResNet structure. There were only 300 hyperspectral images, consequently, only 300 images from each wavelength. Each ResNet classifier had an input dataset of 300, which was split into training, validation, and test datasets. The validation and test datasets were 10% of the size of the overall dataset, so they contained 30 images each. A dataloader was used to load and shuffle the data before they were split into different datasets, as well as to split the training data into batches of size 60.

Before the data were passed into ResNet, they were pre-processed and augmented through transformations. The data were normalised to ensure that the data had a mean of zero and a variance of 1, helping the model to converge faster.

A range of transformations were used for training the ResNet model, ensuring that the images had the same input size and background information. While the CenterCrop transformation ensured the images only contained pistachios, the RandomCrop transformation chose a random area of the cropped images for improved generalisation. The RandomRotation technique additionally enhanced the data and generalisation by rotating images of a random array of pistachio nuts. These modifications increased the variety of the training data, helping the models to not overfit to the training data.

#### 5.2.2. Residual Architecture

The Residual Network Block featured two convolutional layers, each followed by batch normalisation and ReLU. Both convolutional layers had a 3 × 3 kernel, which are commonly used in architectures capturing spatial features [35]. The first convolutional layer used a stride of 2 and a padding of 1. The second convolutional layer used a stride of 1 and a padding of 1 to ensure that the output size was maintained. The batch normalisation accelerated training and increased regularisation by normalising activations to have a mean of 0 and variance of 1. ReLU introduced non-linearity into the model, enabling it to learn increasingly complex patterns. ReLU also set any activations less than zero to zero, speeding up the learning process by effectively turning off these activations.

**Figure 18:**
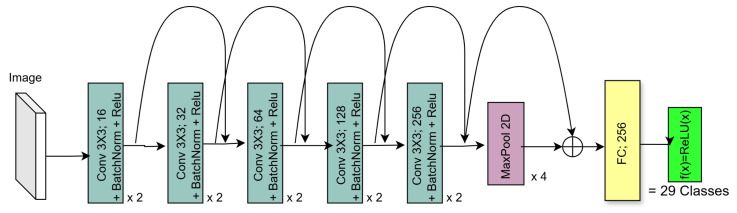
Implemented ResNet Block with ReLU activation functions.

The input passed through the convolutional and batch normalisation layers as well as the first ReLU activation. Then, the output of this was added to the input using the shortcut connection. The result of this was then passed into the final ReLU activation function. The overall ResNet Block architecture implemented for this article can be seen in Figure 18.

#### 5.2.3. Results

**Figure 19:**
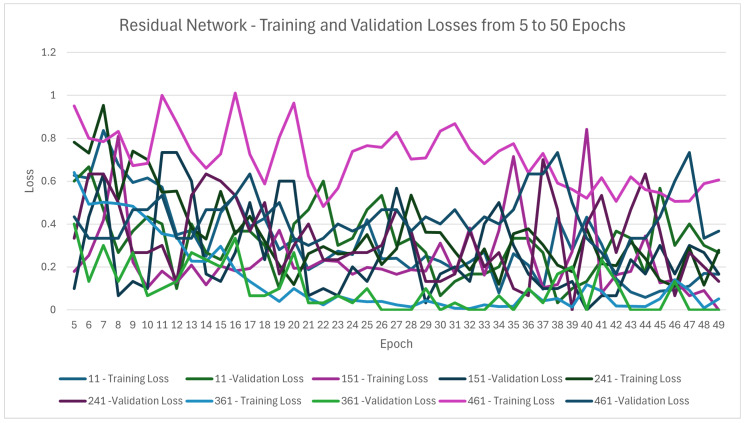
Training and validation losses for each of the ResNet classifiers trained on the dataset of individual wavelength images from bands 11, 151, 241, 361, 461. The classifier trained on the dataset for band 361 on average had the lowest training and validation loss, which also fluctuated less than the loss of the other ResNet classifiers. The graph shows losses from 5 epochs onwards so that the losses are not saturated by the steep learning curve.

The ResNet classifier, as described above, was trained using the wavelength images from each of the five different band levels identified in Section 4.1.3 for 50 epochs. The training and validation losses were recorded after each epoch. The training loss was the cross-entropy of the actual labels and the model’s predictions of the training data in each epoch. The validation loss was 100 minus the score of the classifier on the validation dataset, all divided by 100 after each epoch. The training and validation losses for each of the ResNet classifiers trained on the dataset of individual wavelength images from bands 11, 151, 241, 361, and 461 can be seen in Figure 19.

Using Table 3 and Table 4, it was trivial to see that the validation set had an accuracy = precision of each level = recall of each level = F1-score of each level = 100%. Using Table 5, the test dataset’s evaluation metrics were defined as follows:

These evaluation metrics were very high, indicating that the classifier using images from band 361 could successfully classify the aflatoxin level within a sample of pistachios. The band 361, which corresponds to wavelength 866.21 nm, had a higher accuracy on both the validation and test datasets than any of the other bands tested. This suggested that the wavelength 866.21 nm showed the aflatoxin the most out of the five wavelengths used to train the classifiers.

### 5.3. Variational Autoencoder

A Variational Autoencoder (VAE) was implemented in Python (version 3) using the torch package, as described in Section 3.2, with an encoder and a decoder, using the KL divergence and a reconstruction loss.

#### 5.3.1. Structure of the Encoder and Decoder Networks

**Figure 20:**
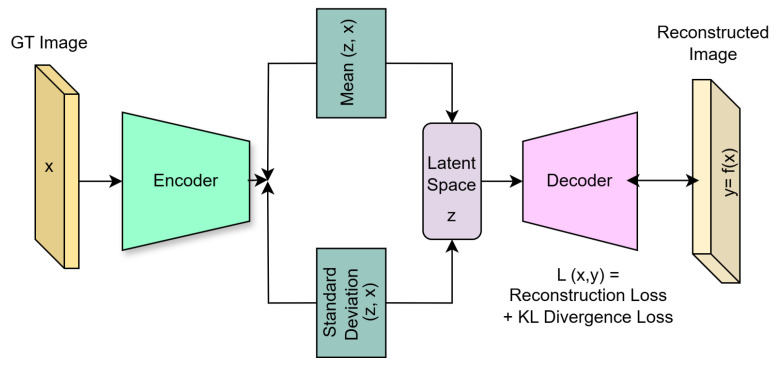
Implemented VAE architecture.

The encoder network generates the latent space representation of an input picture using two convolutional layers. With a kernel size of three, stride of two, plus padding, the layers minimise the input’s spatial dimensions by half while retaining significant spatial characteristics. In order to capture more complex details, the layers extend the number of channels. A ReLU layer brings non-linearity into the model, strengthening its ability to capture complex information and speeding up computation. In order to produce vectors for the mean and log variance of a latent variable distribution of the input image, the output is flattened, passed into a fully connected layer, ReLU, and finally, into another fully connected layer. Using the encoder’s reverse framework, the decoder network produces a reconstructed image. A final function, called the sigmoid activation, is used to normalise the output after it has been transformed into a vector and run via transpose convolution, ReLU, and additional transpose multiplication. Our designed VAE framework is shown in Figure 20.

The model also used the reparameterisation trick, described in Section 3.2, to allow for backpropogation when sampling from the latent space. This was important because sampling was not differentiable; so, in order to backpropogate, the reparameterisation trick approximated the sampling in a differentiable way.

#### 5.3.2. β-Variational Autoencoder (β-VAE)

**Figure 21:**
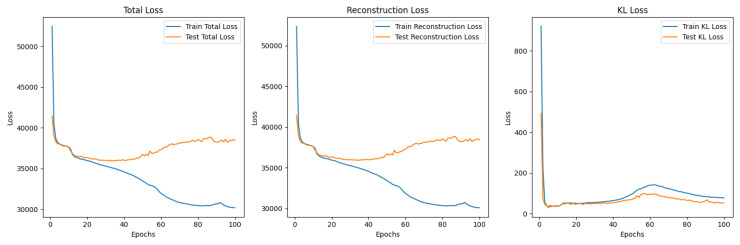
Loss functions for the dataset of band 151 images using β=0.1 to β=1 for 100 epochs. The training loss for the Total Loss and Reconstruction Loss continue to decrease while the test loss for both increases after approximately 25 epochs. This indicates that the model is overfitting. The KL Divergence for both the training and test dataset appears to be slowly increasing, then at approximately 60 epochs, they begin decreasing. The Total and Reconstruction Loss curves appear similar to one another but the KL Divergence loss curves do not.

A β was added to the overall loss function to weight the KL Divergence loss compared to the reconstruction loss: TotalLoss=ReconstructionLoss+β∗KLDivergence. This model is called a β-VAE. The β initially started at 0.1 and was incremented until the final epoch which had a β=1. However, these values, when run for 100 epochs, put too much of an emphasis on the reconstruction loss, leading to the model overfitting to the training data, as seen in Figure 21. This was because the learning focused on being able to accurately reconstruct images from the latent space representations, whereas the KL divergence helps to regularise the latent space so that it is able to generate comparable new samples.

**Figure 22:**
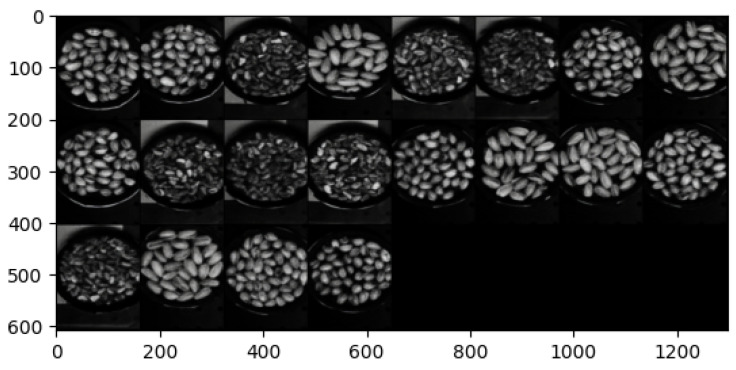
Reconstructed images for the training dataset of band 151 images using β=0.1 to β=1 for 100 epochs. The images are reconstructions of images from each of the 3 aflatoxin levels.

**Figure 23:**
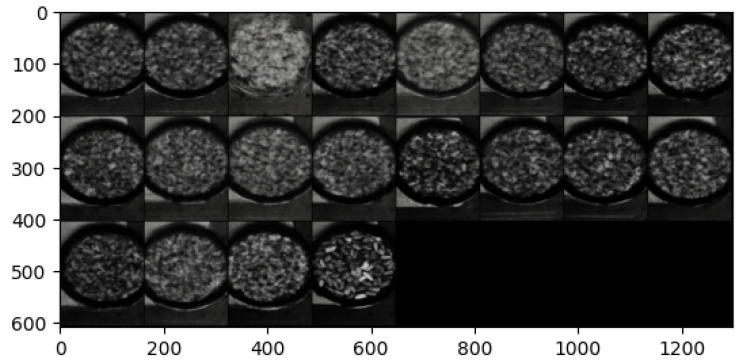
Reconstructed images for the test dataset of band 151 images using β=0.1 to β=1 for 100 epochs. These images are all reconstructions of pistachios without shells from the Greater Than 160 ppn dataset.

It is clear to see that the training reconstructions in Figure 22 are much more well defined and accurate than the reconstructions of the test dataset in Figure 23. This shows that the model was overfitting to the training data.

**Figure 24:**
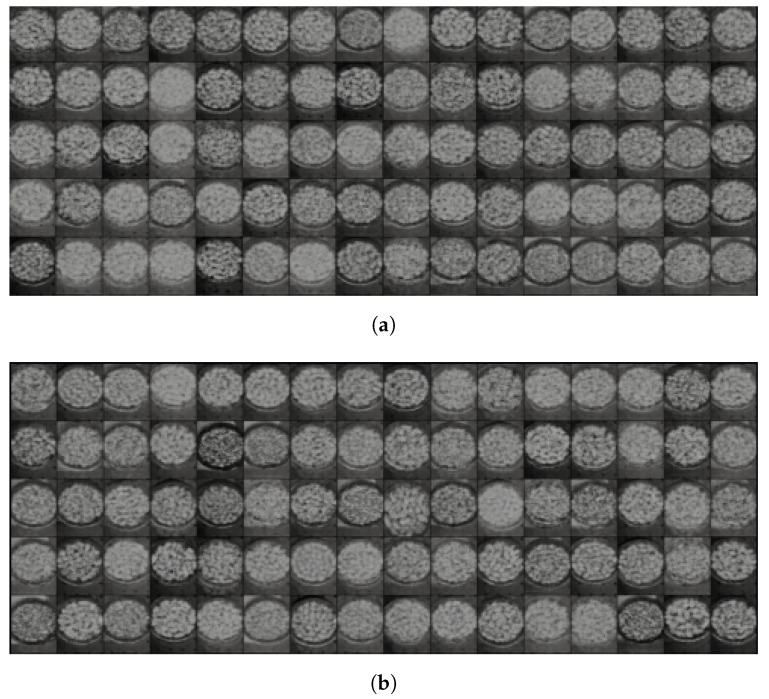
Generated image samples for both the training and test datasets after training the β-VAE using an incremental β from 0.1 to 1 over 100 epochs on the input dataset of band 151: (**a**) Generated images for the training dataset of band 151 images using β=0.1 to β=1 for 100 epochs. (**b**) Generated images for the test dataset of band 151 images using β=0.1 to β=1 for 100 epochs.

Both of the generated samples of images in Figure 24a,b are not good representations of the input data. They are very blurry and it is not even possible to distinguish whether a generation is of pistachios with or without shells. This indicates that the latent space was not well defined and continuous. To visualise the latent space of the trained model, t-SNE was implemented as described in Section 3.2.

**Figure 25:**
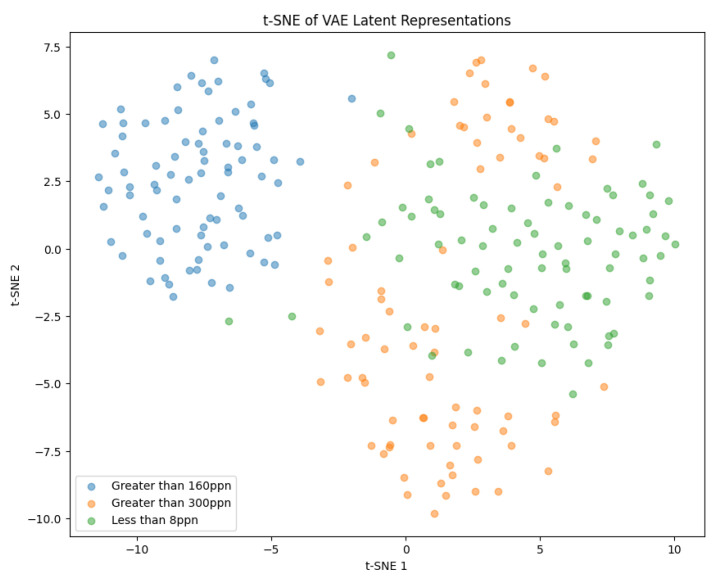
Two-dimensional t-SNE representation of the latent space for the training data after training the β-VAE using an incremental β from 0.1 to 1 over 100 epochs on the input dataset of band 151.

The latent space in Figure 25 is able to distinguish the Greater Than 160 ppn images accurately, which is shown by the separable cluster in the graph. This makes sense because these images contained pistachios without shells, whereas the other images contained pistachios with shells. The clusters for the Less Than 8 ppn and Greater Than 300 ppn levels have a lot of overlap, suggesting that the model had not produced a well-defined latent space which could distinguish between these levels.

The images also contained the backgrounds and bowls which could be affecting the results and distracting the VAE model from learning the features of the pistachios.

#### 5.3.3. β-VAE Redesigned

In order to solve the overfitting problem and to improve the latent space, the β was adjusted to be incremental between 1 and 10. The batch size was also increased, settling on 100 after rigorous testing of various batch sizes. The model was run for 200 epochs to ensure these changes improved performance and reduced the overfitting. The images were also pre-processed to remove the background and the bowl to allow the model to focus its learning on the pistachios. This pre-processing was performed using the CenterCrop and RandomCrop transformations.

**Figure 26:**
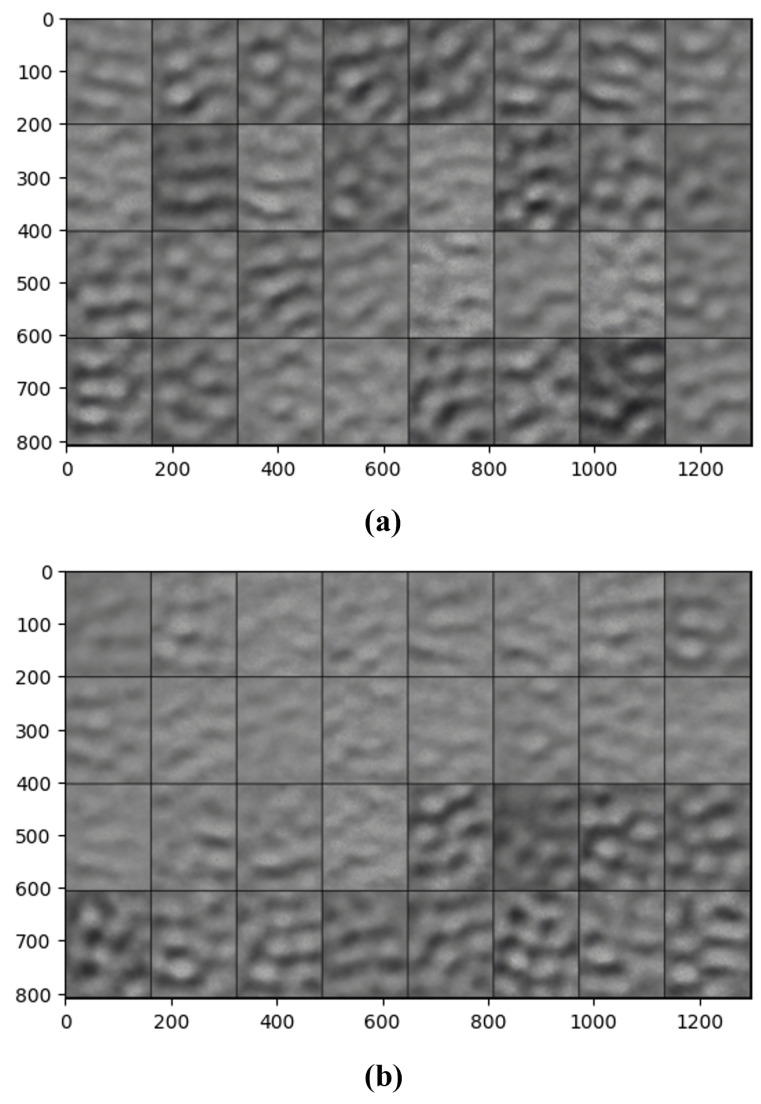
Reconstructed images for the training and test datasets of band 361 images using β=1 to β=10 for 200 epochs. (**a**) Reconstructed images for the training dataset, representing each of the 3 aflatoxin levels. (**b**) Reconstructed images for the test dataset, representing the Greater Than 160 ppn level.

**Figure 27:**
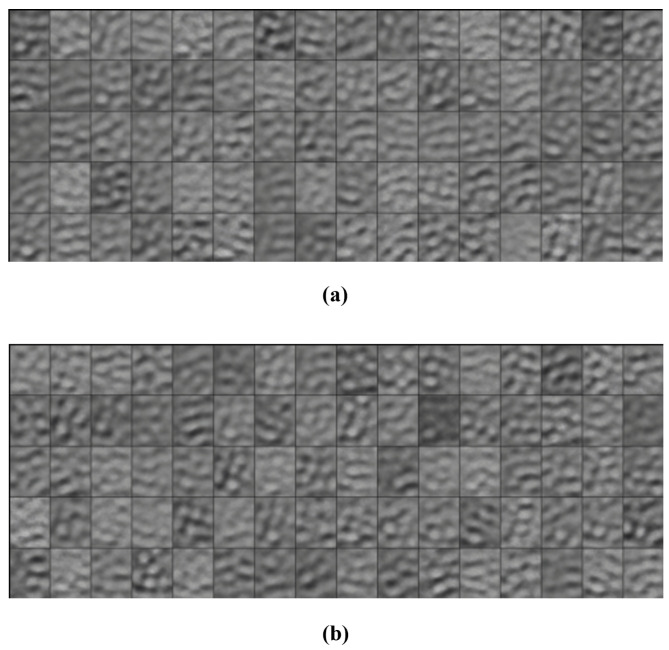
Generated image samples for both the training and test datasets after training the β-VAE using an incremental β from 1 to 10 over 200 epochs on the input dataset of band 361: (**a**) Generated images for the training dataset, and (**b**) Generated images for the test dataset.

**Figure 28:**
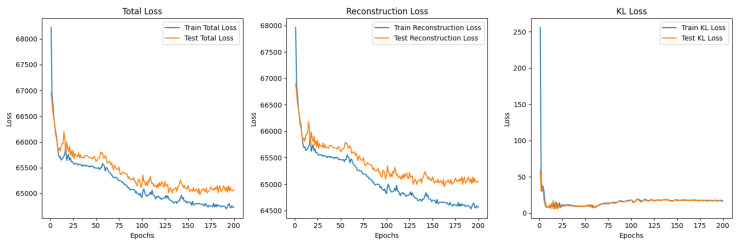
Loss functions for the dataset of band 361 images using β=1 to β=10 for 200 epochs with batch size 100. The training and test loss for the Total Loss and Reconstruction Loss decrease. The KL Divergence for both the training and test dataset appear to be increasing at a decreasing rate, indicating that they may begin the decrease and converge to a loss of 0.

**Figure 29:**
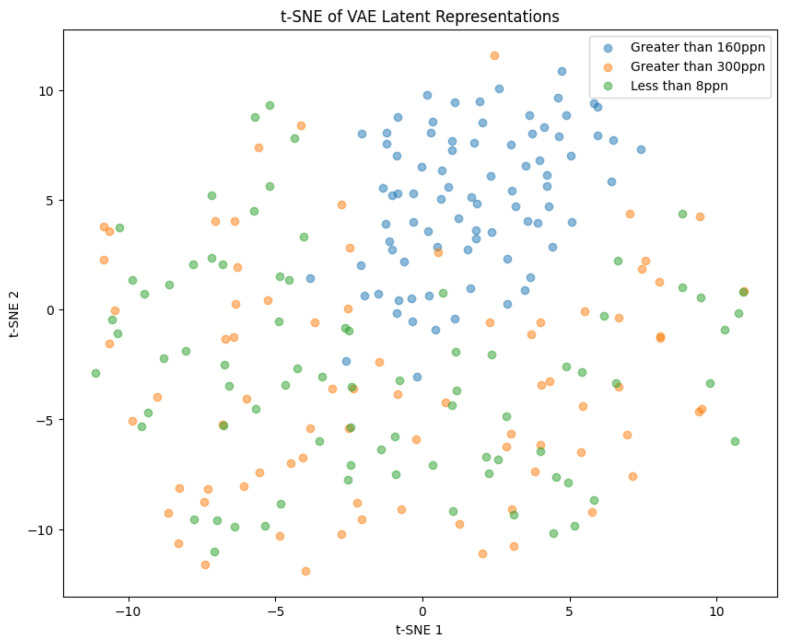
Two-dimensional t-SNE representation of the latent space for the training data after training the β-VAE using an incremental β from 1 to 10 over 200 epochs on the input dataset of band 361.

Although, the model was not yet able to accurately reconstruct or generate images of pistachios, the images produced for the training and test datasets in Figure 26a,b and Figure 27a,b, are similar to one another. This indicates that the model was not overfitting. The loss functions, in Figure 28, indicate positive signs that with longer training the losses would continue to decrease and the model would be able to accurately reconstruct and generate images. The t-SNE representation of the latent space in Figure 29 showed the Greater Than 160 ppn cluster is well defined but the Less Than 8 ppn and Greater Than 300 ppn clusters still have a lot of overlap. With more data and training for more epochs, it is likely that as the KL Divergence decreases these two clusters would become more separable.

The developed VAE model could also be used to generate new image samples to expand the dataset further. The input data were discrete from specific levels; therefore, a well-defined latent space could be used to generate samples which lie between these discrete levels, e.g., between 8 and 160 ppn. This could allow a model to produce more specific aflatoxin level results, not just the discrete level the image lies in.

### 5.4. Deep Convolutional Generative Adversarial Network

#### 5.4.1. Data Pre-Processing

Initially, a Deep Convolutional Generative Adversarial Network (DCGAN) model was implemented as described in Section 3.3, using Python’s torch package and the original wavelength images as inputs without any cropping. The images were normalised and resized to speed up the training. The dataset of size 300 was split into batches of 48 and the model trained the generator and discriminator networks for 100 epochs.

#### 5.4.2. Proposed DCGAN Structure

**Figure 30:**
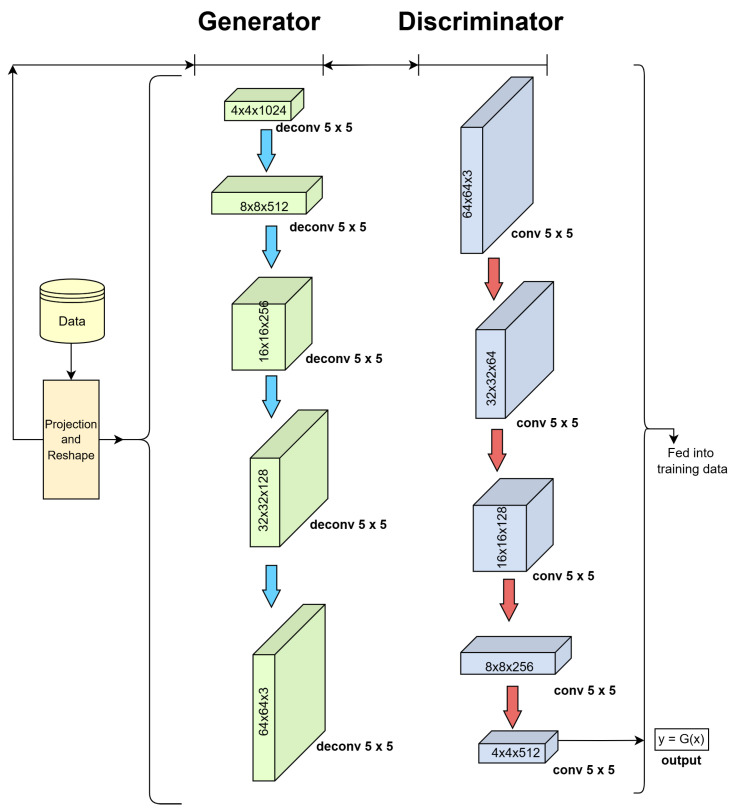
Implemented DCGAN architecture.

The generator network uses a low-dimensional latent vector to generate a high-dimensional image, similar to the VAE model. Following reshaping, the vector is sent across five transposed convolutional layers, each of which has batch normalisation and ReLU. ReLU provides non-linearity, permitting the network to store complex patterns. Batch normalisation helps to stabilise training by normalising each layer’s output, and for every pixel, the TanH function returns −1 to 1. The discriminator network determines if an image is generated or real. Except for the last layer, it consists of six convolutional layers, each featuring batch normalisation and LeakyReLU function. The convolutional layers progressively reduce the spatial dimensions of the input image, increasing its number of channels until the output is a single value. The sigmoid function classifies it by providing a probability score that ranges from 0 to 1. LeakyReLU maintains GAN training by avoiding the dying ReLU problem. Our proposed DCGAN architecture is shown in Figure 30.

#### 5.4.3. Challenges of Using DCGANs

**Figure 31:**
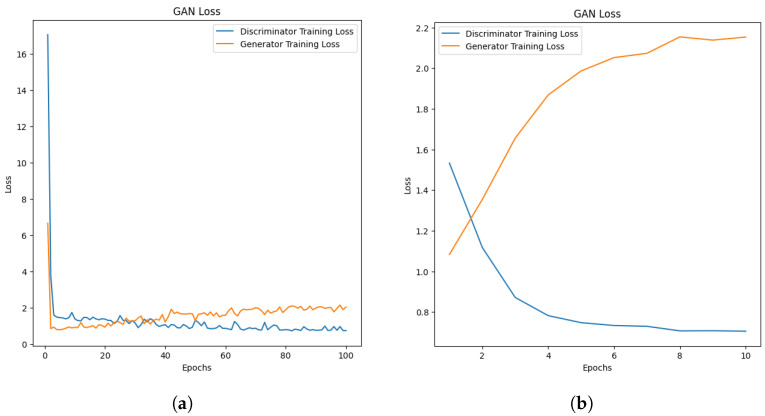
Training losses for both DCGAN models implemented: (**a**) Discriminator and generator training loss for a DCGAN model trained on 100 epochs using the image dataset of band 11. The discriminator loss gradually decreases; meanwhile, the generator loss gradually increases. (**b**) Discriminator and generator training loss for a DCGAN model trained on 10 epochs using a dataset of each wavelength image from 8 hyperspectral images from each of the Less Than 8 ppn and Greater Than 300 ppn levels. The discriminator loss decreases; meanwhile, the generator loss increases.

The main challenge faced when using DCGANs is based on the size of the datasets used. Initially, an attempt was made to train the DCGAN using each identified individual wavelength dataset, which only contained 300 images with very little variety. However, the discriminator was easily able to distinguish which images were generated and the generator was unable to learn how to fool the discriminator, as seen in Figure 31. The initial plan had been to train the DCGAN on a dataset from each of the five bands; however, it was clear that with a dataset of this size, this would not be possible.

To tackle this challenge, the size of the dataset was increased to contain 7392 wavelength images from all bands, this time only distinguishing between the pistachio images with shells. The additional wavelength images increased both the variety and size of the dataset. The new model was used to evaluate whether it would be possible to use the DCGAN model to classify the images. A batch size of 128 was used for this new model, a commonly used value [43], and the model was trained for 10 epochs, due to the large size of the dataset. The discriminator within the new model was still able to easily detect which images were real and generated, as seen in Figure 31a,b. The results indicate that a much larger dataset would need to be used in order to effectively train a DCGAN model.

It was not possible to conclude whether it is possible to train a DCGAN model to detect aflatoxin in a similar manner to which Tan et al. [44] detected pesticide residues from hyperspectral images of Hami melon. With a larger dataset, this approach should be explored further, or with the addition of generated images from a successful VAE model.

## 6. Discussion

The goal of this article was to explore the feasibility of using hyperspectral imaging within the pistachio industry to assess aflatoxin levels. We were able to explore multiple novel approaches and develop an accurate classifier using the small dataset, and to investigate which individual wavelengths contained the most information for the implementation of a multispectral camera. All previous evaluations had been undertaken using pixel-wise labelled hyperspectral images of pistachios, whereas this paper explored the application of machine learning methods to hyperspectral images of pistachios which were only labelled as a whole.

A successful classifier was developed for whole hyperspectral images, using the unsupervised learning methods Dimensionality Reduction with K-Means Clustering to achieve an accuracy of 84.38%. This indicates it would be feasible to develop a classifier using Dimensionality Reduction with K-Means Clustering which could accurately classify the pistachios aflatoxin level discretely. Combining Dimensionality Reduction with K-Means Clustering and only using a cropped spectral image with 10 wavelengths reduced the time complexity of the K-Means algorithm by at least 34,151.04 times. The classifier was developed using the proportion of colour 6 in the clustered images. The accuracy of this classifier could be improved through further development with a larger dataset and a classification metric using the proportions of multiple colours in the clustered images.

Using Residual Networks it was possible to develop classifiers which took in images from only an individual wavelength and were able to successfully classify their aflatoxin level. The classifier trained using band 361, corresponding to wavelength 866.21 nm, performed the best with a validation accuracy of 100% and a test accuracy of 96.67%. This indicated that it could be feasible for a multispectral camera around wavelength 866.21 nm to be introduced and used in combination with a Residual Network to classify the aflatoxin level of pistachios. This is a much cheaper alternative to a hyperspectral camera and would also produce smaller images, which are quicker to process.

The Variational Autoencoder showed promising results for individual wavelength datasets, such that with further development, the model could be used for aflatoxin detection by detecting anomalies, though this would require a larger dataset and longer training. Due to the size of the dataset, it was not possible to train a Deep Convolutional Generative Adversarial Network model which could accurately generate images of the pistachios; however, with more data, this is an approach which should be explored further.

This article is a feasibility study for further research with the hope of implementation within the pistachio industry. This paper recommends using the following machine learning techniques to develop and evaluate classifiers using hyperspectral data: Dimensionality Reduction with K-Means Clustering and a Residual Network with individual wavelength images. The exploration of generative models should continue. The VAE model showed promising results that with a larger dataset and longer training it could be used for anomaly detection or to generate new images to expand the dataset further. With a larger dataset of hyperspectral images and more time, it would be possible to develop more accurate classifiers than in this paper.

## Figures and Tables

**Table 1:** Performance metrics.

Metric	Value
Accuracy	84.38%
Precision	84.21%
Recall	88.89%
F1-Score	86.49%

**Table 2:** Confusion matrix of the K-Means Clustering classifier using the proportion of colour 6. If the proportion is greater than 11.7%, the image is classified as having an aflatoxin level of Less Than 8 ppn; if it is below 11.7%, the image is classified as having an aflatoxin level of Greater Than 300 ppn.

		True Aflatoxin Level		Total
		Less Than 8	Greater Than 300	
**Prediction**	**Less Than 8**	16	3	19
	**Greater Than 300**	2	11	13
**Total**		18	14	32

**Table 3:** The confusion matrix of the validation dataset from band 361 using the ResNet Classifier trained with wavelength images from band 361 for 50 epochs with batch size 60.

		True Aflatoxin Level			Total
		L8	G160	G300	
**Prediction**	**L8**	10	0	0	10
	**G160**	0	10	0	10
	**G300**	0	0	10	10
**Total**		10	10	10	30

**Table 4:** The confusion matrix of the test dataset from band 361 using the ResNet Classifier trained with wavelength images from band 361 for 50 epochs with batch size 60.

		True Aflatoxin Level			Total
		L8	G160	G300	
**Prediction**	**L8**	10	0	0	10
	**G160**	0	9	1	10
	**G300**	0	0	10	10
**Total**		10	9	11	30

**Table 5:** Corresponding performance metrics.

Metric	Value
Accuracy	96.67%
Precision L8	100%
Precision G300	100%
Precision G160	90%
Recall L8	100%
Recall G160	100%
Recall G300	90.91%
F1-Score L8	100%
F1-Score G160	94.74%
F1-Score G300	95.24%

## Data Availability

A subset of the dataset can be downloaded from: https://doi.org/10.5281/zenodo.14213012, accessed on 24 November 2024. The dataset is available for academic research upon request to the corresponding author. The code can be accessed from: https://github.com/lizziesian/pistachio, accessed on 24 November 2024.

## Abbreviations

The following abbreviations are used in this manuscript:

VAE	Variational Autoencoder
ResNet	Residual Network
DCGAN	Deep Convolutional Generative Adversarial Network
TLC	Thin-Layer Chromatography
IARC	The International Agency for Research on Cancer
MTL	Maximum Tolerated Level
HPLC	High-Performance Liquid Chromatography
UV	Ultra-Violet
LIFS	Laser-Induced Fluorescence Spectroscopy
PCA	Principal Component Analysis
LDA	Linear Discriminant Analysis
LRC	Logistic Regression Classification
SVM	Support Vector Machine
HSI	Hyperspectral Imagining
ICA	Independent Component Analysis
ICA-kNN	Independent Component Analysis and a K-Nearest Neighbour classifer
kNN	K-Nearest Neighbours
FDA	Fisher’s Discriminant Analysis
SFS	Sequential Forward Selection
PLS	Partial Least Squares
PLS-DA	Partial Least Squares-Discriminant Analysis
PCA-DA	Principal Component Analysis with Discriminant Analysis
PCA-kNN	Principal Component Analysis-based K-Nearest Neighbours
CART	Classification and Regression Tree
CNN	Convolutional Neural Network
t-SNE	t-Distributed Stochastic Neighbor Embedding
FP	False Positive
FN	False Negative
TP	True Positive
TN	True Negative
SSH	Secure Shell Protocol
SNR	Signal-To-Noise
OSP	Orthogonal Space Projection
